# Total alkaloids of *Corydalis saxicola* Bunting ameliorate ulcerative colitis through regulation of metabolite networks and gut microbiota

**DOI:** 10.3389/fphar.2025.1721116

**Published:** 2025-12-16

**Authors:** Shuyi Jin, Xinyi Cheng, Feng Han, Qien Li, Jing Shang, Guoyong Xie, Minjian Qin

**Affiliations:** 1 Resources Science of Traditional Chinese Medicines Department, China Pharmaceutical University, Nanjing, China; 2 Chongqing Medicinal Plant Cultivation Institute, Chongqing, China; 3 Tibetan Medical Institute, Qinghai University, Xining, China; 4 Jiangsu Key Laboratory of TCM Evaluation and Translation Institute, China Pharmaceutical University, Nanjing, China

**Keywords:** *Corydalis saxicola Bunting*, ulcerative colitis, linoleic acid, *Lactobacillus*, anti-inflammation

## Abstract

**Background:**

Yanhuanglian (YHL), derived from the dried herb of *Corydalis saxicola* Bunting, can inhibit diarrhea and alleviate bleeding in traditional Chinese medicine. YHL is used to treat dysentery and hematochezia, which are recognized as ulcerative colitis (UC) in traditional Chinese medicine. However, the effectiveness and mechanisms of YHL treating UC remain largely unknown. This study aimed to reveal anti-colitis effect and mechanisms of YHL’s total alkaloids (YTA) against UC.

**Methods:**

Three graded doses of YTA were introduced to DSS-induced colitis mice for 7 days to evaluate the anti-colitis effect. Colon, serum, and fecal untargeted metabolomics were applicated to analyze the differential metabolites. 16S rRNA sequencing was used to analyze changes in the gut bacteria, while gut microbiota depletion and fecal microbiota transplantation further verified the effects of gut microbiota. *Lactobacillus* spp. isolated from the mice feces were screened based on the enrichment abundance of YTA *in vivo* and *in vitro*, and the therapeutic effect of *Lactobacillus johnsonii* enriched with YTA was evaluated in colitis mice.

**Results:**

YTA alleviated weight loss, diarrhea, and hematochezia in colitis mice, reducing inflammation and oxidative stress while restoring intestinal barrier impairment. Untargeted metabolomics profiling of colon, serum, and feces demonstrated that YTA restored the disrupted metabolite profiles, with linoleic acid consistently identified as a key differential metabolite. Through the pathway enrichment, linoleic acid metabolism pathway was highlighted. YTA also ameliorated imbalance of the gut microbiota by significantly increasing the abundance of *Lactobacillus*. Gut microbiota depletion and fecal microbiota transplantation confirmed that the benefits of YTA depended on the presence of gut microbiota. Furthermore, *Lactobacillus johnsonii* enriched by YTA protected colitis mice against UC.

**Conclusion:**

YTA exhibited potential anti-colitis activity by modulating metabolomic profiles and the gut microbiota, suggesting its potential as a complementary and alternative therapy in phytomedicine.

## 1 Introduction

Ulcerative colitis (UC) is an immune-mediated disease that primarily affects the rectum, colonic mucosa, and submucosa (Zhou et al., 2021). UC is a prevalent disease in North America and Europe, reports have shown the recent incidence rates of UC are about 0.024% and 0.019%, respectively (Wang et al., 2022), and the occurrence rate of UC has witnessed an upward trend over the past decade (Qian and Wu, 2018). This rising incidence has heightened the burden of the disease, indicating that UC has become a major health issue in the world. Current pharmaceutical therapies for UC mainly include aminosalicylic acids, glucocorticoids, immunosuppressors, and biologicals, all of which have certain side effects, such as inducing gut microbiota dysfunction, metabolic disorders, liver and kidney impairment, and anaphylactic reactions (Le Berre et al., 2023). Therefore, the exploration and development of traditional Chinese medicine (TCM) for UC treatment have great significance, based on its advantages of multiple components, multiple targets, and holistic regulation.

The pathogenesis of UC is closely associated with metabolic disorders and gut microbiota dysfunction, which interact and jointly induce inflammation and oxidative stress, and further destroy the intestinal epithelial barrier (Kaser et al., 2010). Metabolic disorders involving in long-chain fatty acids (LCFAs), are another key feature in colitis patients. The metabolic balance of LCFAs is closely linked to intestinal homeostasis, and the metabolic disorders of LCFAs are considered a potential driver. Studies have shown that the LCFA contents in the serum are markedly elevated in UC patients compared with healthy individuals, with a significant increase in stearic acid, palmitic acid (PA), oleic acid, and linoleic acid (LA) (Wei et al., 2024). Current research indicates that LCFAs can promote the development of UC by disrupting intestinal integrity, affecting the gut microbiota, and activating inflammatory pathways (Adolph et al., 2022). Gut bacteria constitute the majority of the gut microbiome, and an imbalance of gut bacteria is characterized by an increased ratio of potential pathogenic bacteria to beneficial bacteria and a decreased diversity. Clinical studies have demonstrated that the abundance of *Enterococcus* spp., *Streptococcus anginosus*, *Ruminococcus gnavus*, *Escherichia coli* significantly increases in colitis patients, while *Lactobacillus* spp., *Bifidobacterium longum*, *Faecalibacterium prausnitzii*, and *Roseburia intestinalis* significantly decrease (Iliev et al., 2025; Guo P. et al., 2024). Collectively, metabolic disorders and gut microbiota imbalance jointly drive UC, and holistic regulation of TCM can target this complicated network.

The whole plant of *C. saxicola* Bunting (*Corydalis saxicola*) is a common TCM, known as Yanhuanglian (YHL). YHL is a perennial herbaceous plant belonging to the genus *Corydalis* in the family Papaveraceae. In TCM theory, YHL is bitter in taste and cold in nature, tropistic to the Stomach and Large Intestine Meridians, with core effects of clearing heat, detoxifying, eliminating dampness, relieving pain, and stopping bleeding. Its medicinal value has been documented in *Dian Nan Ben Cao* (Materia Medica of Southern Yunnan) (Lan, 2017) during the Ming Dynasty, which records its efficacy in treating sore throat, resolving abscesses, and relieving pain, and also notes that YHL soaked in milk is effective for treating pinkeye. *Zhong Hua Ben Cao* (Chinese Materia Medica) (National Administration of Traditional Chinese Medicine, 1999) records that YHL helps with diarrhea, abdominal pain, and dysentery, and notes that YHL steamed with wine can alleviate dysentery which is recognized as UC in TCM. In additional, *Qian Nan Ben Cao* (Guizhou Materia Medica) (Si, 2015) also records that YHL decocted with Euphorbiae Humifusae Herba and Gnaphalium japonicum Herba in water can alleviate hematochezia, which is another condition recognized as UC in TCM. YHL is also widely used in ethnic minority medicine in China. The Yi, Zhuang, and Miao ethnic groups, mainly living in humid Southwest China and preferring spicy food, usually use YHL to treat oral, respiratory diseases, and intestinal diseases. According to *Yun Nan Yi Yi Yao* (Yunan Yi Ethnic Medicine) (Yunnan Province Yi Hospital, 2001), the Yi people use fresh juice of YHL to treat oral ulcers, a decoction of YHL combination with Carpesium divaricatum Herba to treat hyperpyrexia, and a decoction of YHL combination with Melastoma dodecandrum Herba to treat colitis. According to the *Zhong Guo Zhuang Yao Xue* (Chinese Zhuang Ethnic Medicine) (Zhong et al., 2016), the Zhuang people take YHL internally combined with Lysimachiae Herba, Phyllanthus urinaria Herba, and Abri Herba to address pneumonia, a decoction of YHL and Evodiae Fructus to alleviate colds, and a decoction of YHL to treat chronic colitis. *Zhong Guo Miao Zu Yi Xue* (Chinese Miao Medicine) (Tian, 2013) records that the Miao people use YHL decoction in water to treat abdominal pain, hematochezia, and dysentery. And YHL ensures a stable supply at relatively lower costs, as it is widely distributed in Southwest China with well-developed artificial cultivation techniques, which can effectively reduce financial burden of patients and features both clinical practicality and economic benefits. The major active components of YHL are total alkaloids (YTA), including dehydrocavidine, berberine, and sanguinarine. Among them, sanguinarine has antioxidant effects (Niu et al., 2013), while berberine and palmatine have anti-inflammatory effects (Li et al., 2016; Ji et al., 2024). Modern studies have demonstrated YTA have potential therapeutic roles in conditions such as metabolic dysfunction-associated steatohepatitis (Sun et al., 2025), non-alcoholic fatty liver disease (Guo Y. et al., 2024), liver fibrosis (Wang et al., 2023), and pain relief (Xue et al., 2021). However, there are no studies that have confirmed whether YHL can treat UC as recorded in medical classics, and the therapeutic effect and underlying mechanism of YTA in treating UC remain to be elucidated.

This study aimed to explore whether YTA improved UC pathology by specifically regulating the composition of gut bacteria and influencing metabolic profiles. Metabolomics analysis was conducted to identify significant alterations in serum, colon, and feces, while 16S rRNA analysis was performed to assess changes in gut bacteria. Further, gut microbiota depletion and fecal microbiota transplantation (FMT) were used to investigate the relationship between YTA and gut microbiota, and verified whether *Lactobacillus johnsonii* (LJ) enriched by YTA contributed to the anti-colitis effect. This is the first study to confirm the anti-colitis activity of YTA, providing a new direction for the treatment of UC.

## 2 Materials and methods

### 2.1 The preparation of YTA

*C. saxicola* was harvested in Shiyan City, Hubei Province, and the voucher specimen was preserved in the Department of Traditional Chinese Medicine Resources, China Pharmaceutical University with HB1912JSY. YTA was prepared based on previous references (Zeng et al., 2020; Yin et al., 2019; Xie et al., 2021), and analyzed following previously established methods (Xie et al., 2021) (Method S1). The dried powder was suspended in 0.5% CMC-Na aqueous solution to formulate concentrations of 50, 100, and 200 mg/kg, and the dosages were converted based on *Zhong Hua Ben Cao*.

### 2.2 Establishment of UC mouse model induced by DSS

Male C57BL/6J mice (SPF grade, 5–6 weeks, 18–22 g) were purchased from Nanjing Ruixingshanhai Biotechnology Co., LTD (Nanjing, China). A 12-h light/dark cycle was set up and temperature was regulated within 22 °C–25 °C.

After the mice underwent a 7-day adaptation period, they were randomly allocated into 5 groups: control group (Control), model group (DSS), high-dose YTA group (200 mg/kg, DSS + YTAH), middle-dose YTA group (100 mg/kg, DSS + YTAM), low-dose YTA group (50 mg/kg, DSS + YTAL), and 5-aminosalicylic acid (5-ASA) (200 mg/kg, DSS+5-ASA). Except for the control group receiving reverse osmosis water, all other groups had free access to 3% dextran sulfate sodium salt (DSS, 160110, MP Biomedicals) for 7 days. The YTA groups received different concentrations of YTA at a fixed time each day and the 5-ASA group received 5-ASA (5-ASA, A823148, Macklin), while other groups received an equal volume of 0.5% CMC-Na normal saline solution. During the experimental period, body weight, hair condition, stool characteristics, food consumption, mental status, and disease activity index (DAI) were recorded.

### 2.3 Gut microbiota depletion and FMT

A cocktail of antibiotics (ABX) (neomycin sulfate (n814740, Macklin) 1 g/L, metronidazole (m813526, Macklin) 1 g/L, vancomycin (v871983, Macklin) 0.5 g/L, and ampicillin (a105484, Macklin) 1 g/L) was administered to deplete gut microbiota in mice, establishing the pseudo-germ-free (PGF) mouse model. DSS induction and YTA treatment, as described as before, were initiated 7 days after treatment of ABX (Zeng et al., 2020; Li et al., 2021). FMT was followed as described in previous references (Zeng et al., 2020; Li et al., 2021). Donor mice were treated with 3% DSS or YTA (200 mg/kg) and 3% DSS for 7 days. Recipient mice received fresh fecal transplants daily from donor mice. The specific process was shown in Supplementary Figure S1.

Fresh feces were collected from donor mice daily, and diluted with 10 volumes PBS. The supernatant was collected after thorough mixing. Subsequently, the OD_600_ value of the mixed bacterial solution was adjusted to 1. The bacterial solution was diluted twice, and 200 µL was administered orally to each recipient mouse.

### 2.4 Histopathological examination and immunohistochemistry

Colon tissues were immersed in neutral 4% paraformaldehyde, embedded in paraffin, sectioned, and stained with H&E. Immunohistochemical staining was followed the previous protocol (Cecchini et al., 2019). Colon tissues were stained with ZO-1 and occludin antibodies, and the samples were examined under a microscope to record characteristic findings.

### 2.5 Biochemical determination

Colon tissue samples were processed according to the kit instructions. The supernatants were tested for tumor necrosis factor-α (TNF-α), interleukin-10 (IL-10), interleukin-6 (IL-6), interleukin-1β (IL-1β), matrix metalloprotein-9 (MMP-9) (Hangzhou Muti Science, China), and LA (Genmed, China) with ELISA kits. Additionally, they were tested for malondialdehyde (MDA), myeloperoxidase (MPO), and superoxide dismutase (SOD) (Nanjing Jiancheng, China).

### 2.6 Quantitative real-time PCR (qPCR)

The extraction of RNA was based on the manufacturer’s guidelines for the TRIzol method (R401-01, Vazyme), and the RNA was then reverse transcribed to generate complementary DNA (R302-01, Vazyme). qPCR was carried out by utilizing the Hieff qPCR SYBR Green Master Mix (Q341-02, Vazyme) with the primer sequences specified in Supplementary Table S1. The obtained data were calibrated through internal control gene levels, and gene expression was computed through the 2^−ΔΔCT^ method.

### 2.7 Western blot

Western blotting was performed based on previous protocols (Niu et al., 2013). The samples were lysed with RIPA buffer (W062-1-1, Nanjing Jiancheng) added 1% PMSF (W044-1-1, Nanjing Jiancheng) for 30 min. After incorporating loading buffer (W076-1-1, Nanjing Jiancheng), the mixture underwent boiling. SDS-PAGE gel electrophoresis separated protein in tissue, followed by transfer to membrane and blocked with skim milk. The membrane was incubated with primary antibodies (Supplementary Table S2) overnight, washed appropriate times with TBST, and then incubated with secondary antibodies (Supplementary Table S2) in suitable time. The blots were developed using ECL luminescence.

### 2.8 16S rRNA sequencing of fecal microbiota and genomic sequencing analysis of LJ

The metagenomic DNA in feces was isolated through the CTAB method. Illumina TruSeq® DNA PCR-Free Sample Preparation Kit (Illumina, United States) served as construction of the library. Amplification of V3-V4 variable region was carried out with primers 341F and 806R. Sequencing was performed on the NovaSeq 6000 PE250 platform by Weikemo Co., Ltd. (Shenzhen, China) (Bokulich et al., 2018; Callahan et al., 2016). The data analysis included principal component analysis (PCA), principal coordinates analysis (PCoA), linear discriminant analysis effect size (LEfSe), and correlation analysis. And all conducted using the Weikemo bioincloud platform.

Genomic DNA from LJ was extracted and subjected to quality testing. The sequencing library was prepared with Illumina’s TruSeq DNA PCR-free preparation kit. The quality of the library was validated, and high-throughput sequencing was performed at General Biol Co., Ltd. (Chuzhou, China).

The raw data were uploaded to the China National Center for Bioinformation (https://ngdc.cncb.ac.cn) under CRA019815.

### 2.9 Untargeted metabolomics analysis

Metabolites in serum and colon were detected using an Agilent 1290 Infinity LC in combination with a TripleTOF 6600 mass spectrometer (AB Sciex, Foster City, CA, United States), and metabolites in feces were detected with an SCIEX Exion LC in combination with a TripleTOF 5600 mass spectrometer (AB Sciex, Foster City, CA, United States). Results were analyzed with XCMS, Markerview 1.3.0, Simca 14.1, and Microsoft Excel. For more specific methods, referred to Method S2.

### 2.10 Isolation of LJ and decomposition of LA

Fecal samples from colitis mice treated with YTA were mixed with 5 volumes of PBS. The bacterial suspension was plated on MRS plates (HB0384-1, Qingdao Hope) and cultured for 24 h in an anaerobic incubator, single colonies were selected and subjected to 16S rRNA gene amplification with PCR. The results were analyzed at General Biol Co., Ltd. (Chuzhou, China).

The bacterial suspension was adjusted to an OD_600_ value of 1, diluted twice, and supplemented with LA (l812256, Macklin) or YTA under anaerobic conditions. The supernatant was analyzed based on previous report (Zhang et al., 2022; Folch et al., 1957). Additional detailed methods were provided in Method S3.

### 2.11 Cell culture and cell treatment

Caco2 cells (Procell, China) were cultured in DMEM supplemented with 10% fetal bovine serum and 1.0% penicillin-streptomycin solution at 37 °C in a 5% CO_2_ atmosphere. Caco2 cells were seeded at a density of 1 × 10^6^ cells/mL in 12-well plates and allowed to adhere overnight. LA (2 mg/mL) and lipopolysaccharide (LPS 1 μg/mL) were added for 24 h. The cells were then collected to determine gene expression.

### 2.12 Statistical examination

We conducted data analysis with GraphPad Prism 7.0, Origin 2021, SPSS 22.0, and Microsoft Excel. Data were shown in the form of average values accompanied by the standard deviation (mean ± SD). One-way ANOVA, t-tests and Spearman’s correlation analysis were employed to conduct the differential analysis. *P* value <0.05 was assessed to establish statistical significance.

## 3 Results

### 3.1 Chemical compounds analysis in YTA

**FIGURE 1:**
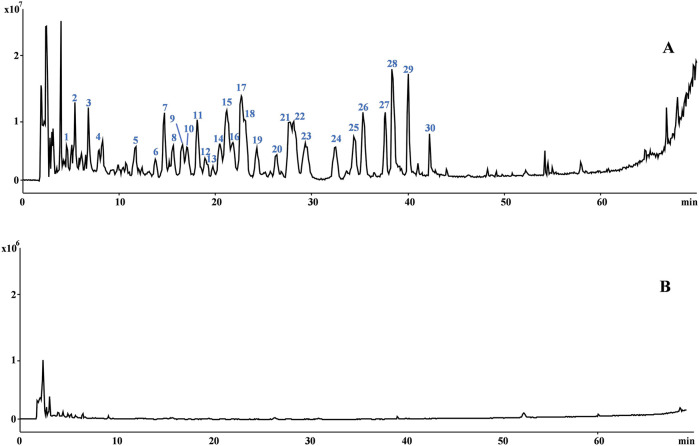
Chemical constitutes in YTA. **(A)** Total ion chromatograms of YTA in positive mode. **(B)** Total ion chromatograms of YTA in negative mode.

The total alkaloids extracted from YHL were identified by HPLC-MS/MS. The total ion chromatogram of YTA was shown in Figure 1, and the chemical compounds were shown in Supplementary Figure S2A and Supplementary Table S3. We identified 30 alkaloids in YTA and quantitative analysis of the main components was presented in Supplementary Figures S2B−E.

### 3.2 YTA treatment effectively alleviated DSS-induced colitis in mice

**FIGURE 2:**
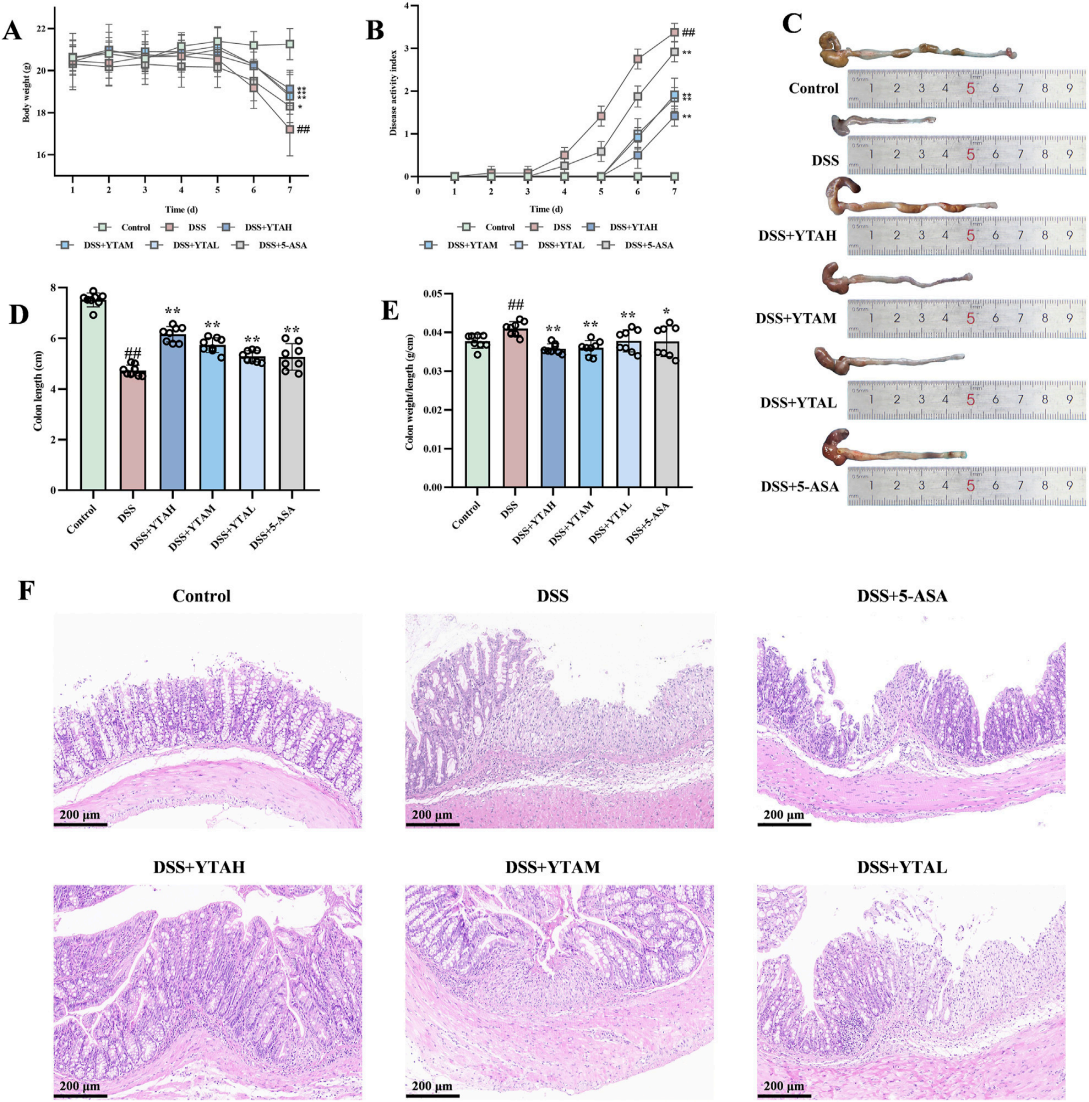
YTA treatment alleviated DSS-induced colitis in mice. **(A)** Changes of body weight. **(B)** Changes of DAI score. **(C)** Representative morphologies of colon. **(D)** Colon length. **(E)** The ration of weight and length in colon. **(F)** H&E staining of colon tissue. **(A**–**E)** n = 8 per group and **(F)** n = 3 per group. Mean values ±SD are presented, *P* values were calculated using One-Way ANOVA, ^##^ *P* < 0.01 compared with control, ^**^ *P* < 0.01 and ^*^ *P* < 0.05 compared with DSS.

In this study, male C57BL/6J mice with DSS-induced colitis received YTA daily at concentrations of 50, 100, 200 mg/kg via oral gavage for 7 days. Three doses of YTA treatment could inhibit weight loss and elevation of DAI scores in colitis mice (Figures 2A,B). Specifically, while colitis led to a 19% reduction in body weight, mice treated with 200 mg/kg YTA only showed a 9% decrease, suggesting that YTA effectively alleviated weight loss. Inflammation in UC led to various degrees of colon lesions with fibrosis, as evidenced by the dull color of colonic tissue in colitis mice with some showing erosion and congestion, while normal colon had a lustrous appearance and rosy color in contrast. YTA treatment could significantly alter lesion changes in the colon, characterized by ameliorating colon shortening and a high colorectal length-to-weight ratio in a dose-dependent manner (Figures 2C–E). Finally, we performed histopathological examination, which revealed a loss of goblet cells, disappearance of intestinal glands, abnormal morphological changes in intestinal epithelial cells, and inflammatory cells infiltrated the colonic region of colitis mice. In contrast, 200 mg/kg YTA treatment significantly inhibited goblet cell loss, reduced intestinal gland disappearance, and alleviated inflammatory cell infiltration in the colon tissue (Figure 2F). Importantly, colitis mice treated with 200 mg/kg YTA exhibited better efficacy than those treated with 5-ASA. Overall, YTA exerted a dose-dependent protective effect against DSS-induced colitis by improving disease symptoms and preserving colon integrity.

### 3.3 YTA treatment alleviated oxidative stress, inflammation, and damaged intestinal barrier in DSS-induced colitis in mice

**FIGURE 3:**
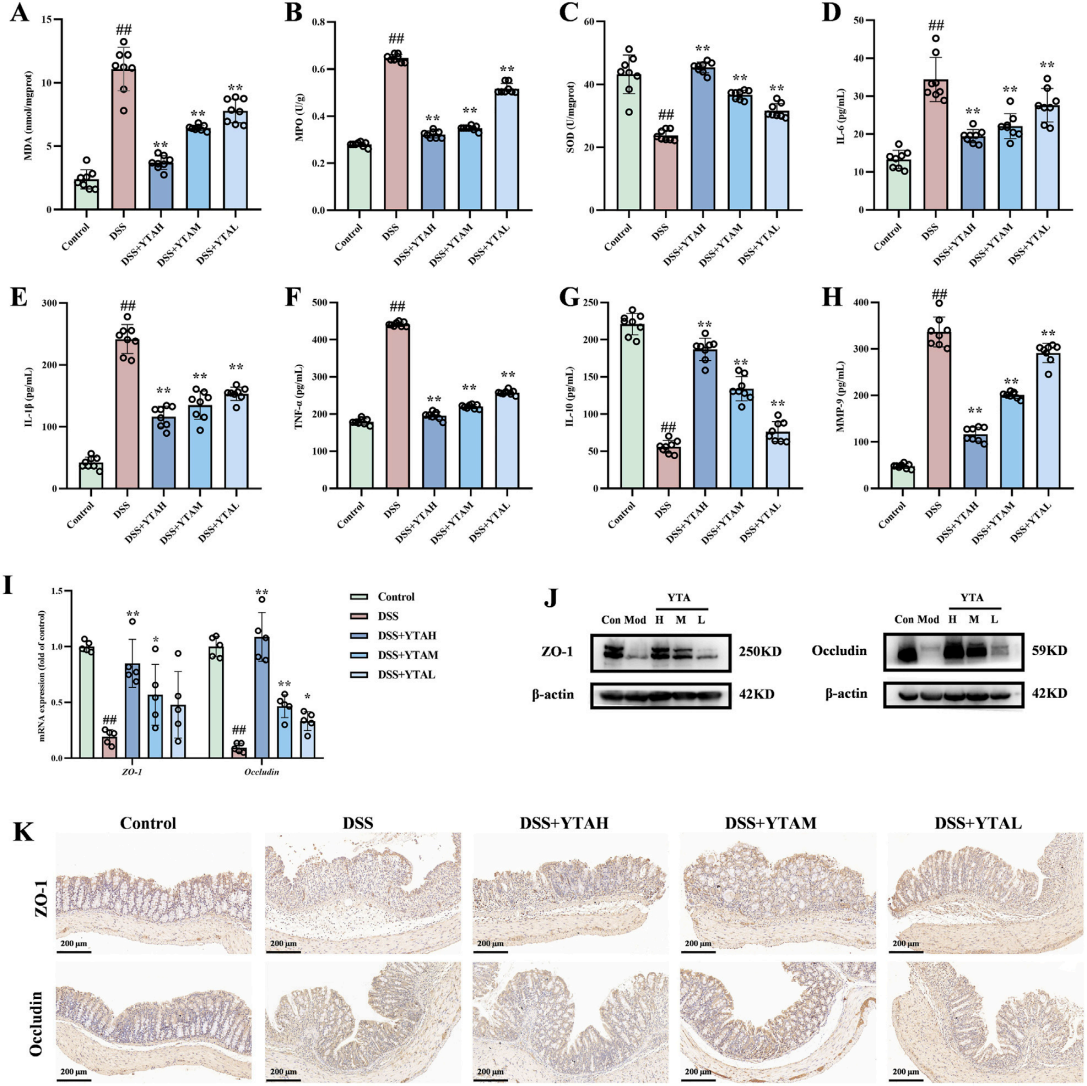
YTA treatment alleviated inflammation, oxidative stress and intestinal barrier injured in colitis mice. **(A)** MDA, **(B)** MPO, **(C)** SOD, **(D)** IL-6, **(E)** IL-1β, **(F)** TNF-α, **(G)** IL-10, and **(H)** MMP-9 contents in colon tissue. **(I)** The mRNA levels, **(J)** The protein expression, and **(K)** Immunohistochemical image of ZO-1 and occludin in colon tissue. **(A**–**H)** n = 8 per group, **(I)** n = 5 per group, and **(J)** and **(K)** n = 3 per group. Mean values ±SD are presented, *P* values were calculated using One-Way ANOVA, ^##^ *P* < 0.01 compared with control, ^**^ *P* < 0.01 and ^*^ *P* < 0.05 compared with DSS.

To further investigate the underlying mechanisms, we detected the antioxidant, anti-inflammatory, and intestinal barrier restoring effects. Throughout this investigation, we found raised concentrations of MDA and MPO in colitis mice compared with control. YTA treatment notably reduced both (Figures 3A,B). SOD, an antioxidant synthesized by the body with various functions, was also restored by YTA treatment (Figure 3C), further enhancing its antioxidant capacity. Moreover, levels of pro-inflammatory cytokines including IL-6, IL-1β, and TNF-α were notably reduced, whereas anti-inflammatory cytokine IL-10 was notably increased after YTA treatment in colitis mice (Figures 3D–G). YTA treatment also substantially decreased levels of MMP-9, a kind of inflammatory mediator (Figure 3H). Additionally, we explored intestinal barrier function. The expression levels showed a dose-dependent trend following YTA treatment (Figures 3I,J). The results indicated that the intestinal barrier damage in colitis mice was improved with the most significant improvements observed at high doses of YTA. Immunohistochemical staining revealed that ZO-1 and occludin proteins were localized on the cell membranes of normal epithelial cells, and YTA treatment helped to repair the damaged intestinal barrier to some extent (Figure 3K). Overall, YTA exerted antioxidant, anti-inflammatory, and intestinal barrier restoring effects in a dose-dependent manner.

### 3.4 YTA regulated colon and serum metabolomic profiles in DSS-induced colitis mice

**FIGURE 4:**
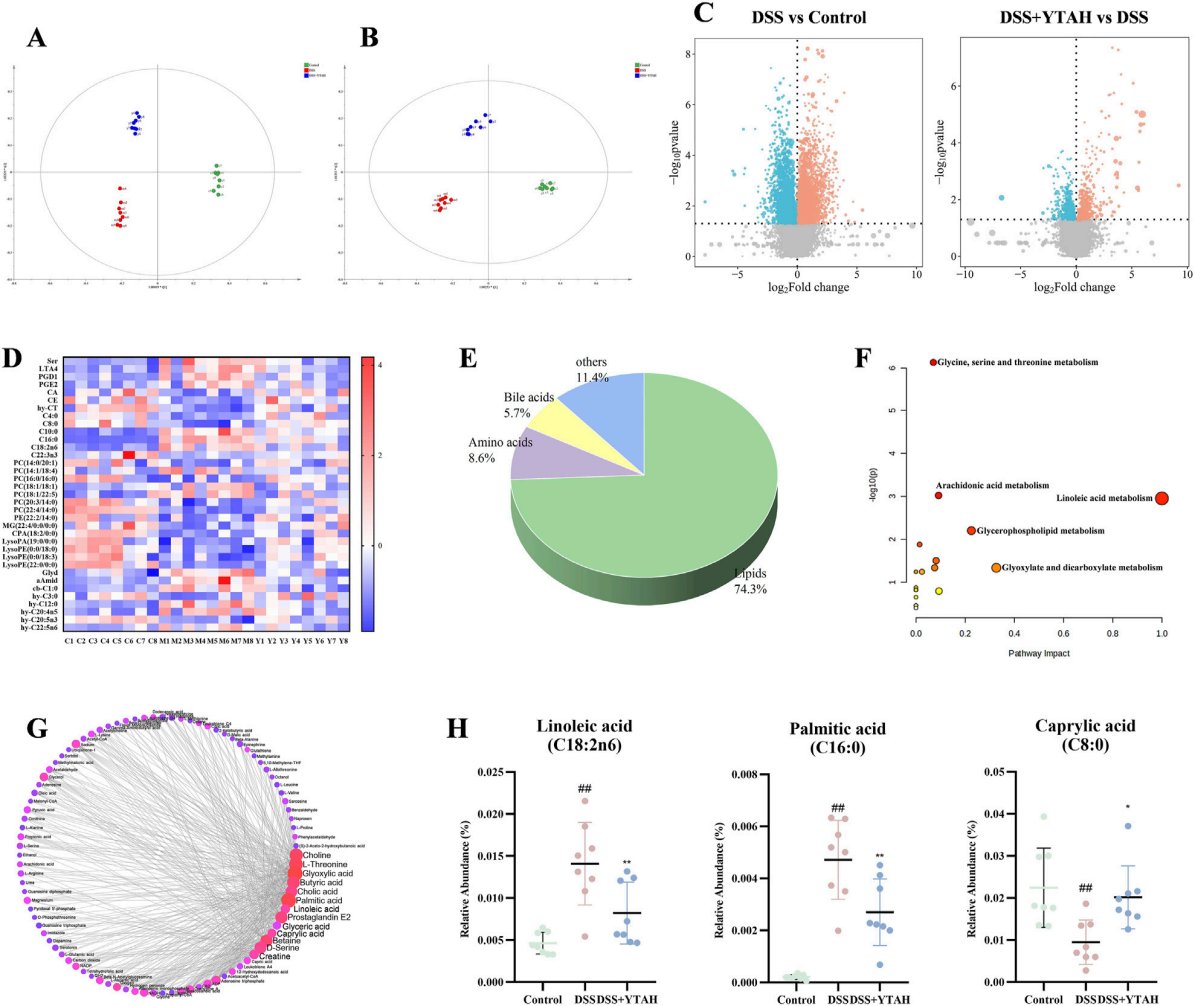
Colon metabolomics analysis of YTA’s therapeutic effect on DSS-induced colitis in mice. **(A)** OPLS-DA analysis in positive mode. **(B)** OPLS-DA analysis in negative mode. **(C)** Volcano plot of metabolites. **(D)** Heat map analysis of significant differential metabolites. **(E)** Types of significant differential metabolites. **(F)** Pathway enrichment of significant differential metabolites. **(G)** Network analysis of significant differential metabolites. **(H)** Abundance of metabolites with a dominant position in both KEGG analysis and network analysis. **(A**–**H)** n = 8 per group. Mean values ±SD are presented, *P* values in **(C)** were calculated using t tests, *P* values in others were calculated using One-Way ANOVA, ^##^ *P* < 0.01 compared with control, ^**^ *P* < 0.01 and ^*^ *P* < 0.05 compared with DSS.

PCA and orthogonal partial least squares-discriminant analysis (OPLS-DA) indicated notable changes existed in metabolites between colitis mice and control mice, and YTA could shift the metabolites of colitis mice toward those of control mice (Supplementary Figures S3A−H, 4A,B). Metabolites were exhibited in volcano plots in which YTA upregulated 434 metabolites and downregulated 530 metabolites compared with colitis mice (Figure 4C). Furthermore, metabolites with VIP >1, |P (corr)| > 0.4, |Log2FoldChange| > 0.5, and *P* < 0.05 were selected as threshold values of metabolite change, and 35 metabolites were screened as differential metabolites (Supplementary Figures S3I, J; Supplementary Table S4). Lipids accounted for 74.3% of differential metabolites (Figures 4D,E). KEGG analysis suggested significant enrichment in glycine, serine and threonine metabolism, LA metabolism, arachidonic acid (AA) metabolism, and glycerophospholipid metabolism, which was in accordance with the above results (Figure 4F). Combined with KEGG analysis and network analysis (Figure 4G), LA was found to occupy a major position and be closely related to other metabolites. YTA could also significantly alter metabolites including LA, PA, and caprylic acid (CA) which occupied key positions in KEGG and network analysis (Figure 4H). Serum metabolomics exhibited consistent results with colon metabolomics, which YTA could restore the metabolomic profile in colitis mice and LA metabolism was the significant enrichment pathway (Supplementary Figures S4A−G; Supplementary Figures S5A−J; Supplementary Table S5). Similarly, KEGG and network analysis indicated that YTA could significantly alter the metabolites such as LA, 13-HPODE, AA, PGD1, PGE1, and PGE2. In conclusion, YTA modified colon and serum metabolites, with a primary regulatory focus on LA.

### 3.5 YTA regulated fecal metabolomics in DSS-induced colitis mice

**FIGURE 5:**
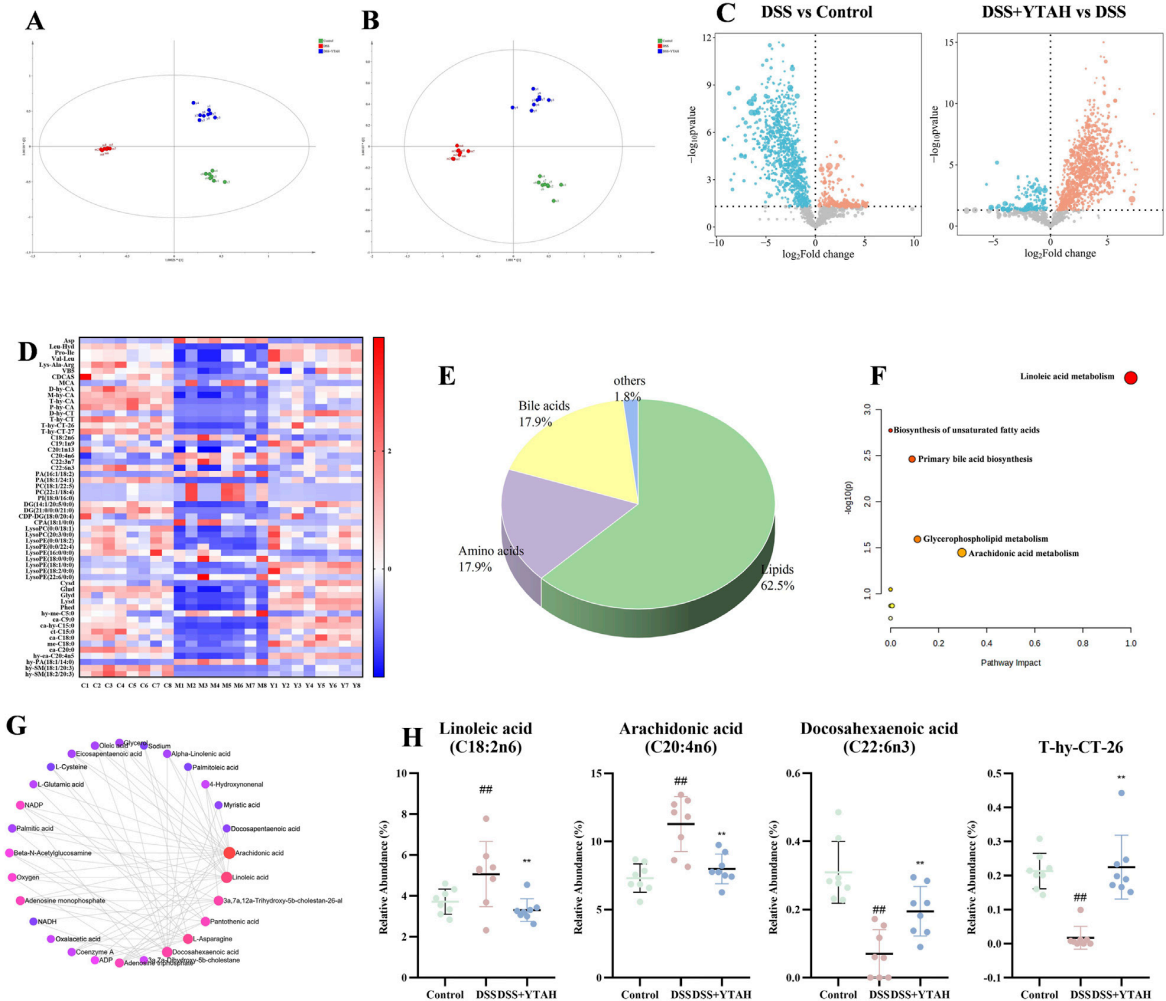
Feces metabolomics analysis of YTA’s therapeutic effect on DSS-induced colitis in mice. **(A)** OPLS-DA analysis in positive mode. **(B)** OPLS-DA analysis in negative mode. **(C)** Volcano plot of metabolites. **(D)** Heat map analysis of significant differential metabolites. **(E)** Types of significant differential metabolites. **(F)** Pathway enrichment of significant differential metabolites. **(G)** Network analysis of significant differential metabolites. **(H)** Abundance of metabolites with a dominant position in both KEGG analysis and network analysis. **(A**–**H)** n = 8 per group. Mean values ±SD are presented, *P* values in **(C)** were calculated using t tests, *P* values in others were calculated using One-Way ANOVA, ^##^ *P* < 0.01 compared with control, ^**^ *P* < 0.01 and ^*^ *P* < 0.05 compared with DSS.

Furthermore, we analyzed the endogenous metabolites in feces. According to PCA and OPLS-DA (Supplementary Figures S5A, B; Supplementary Figures S6A−H), there were significant differences among the control, DSS, and YTAH groups, and distinct clustering existing among the three groups, which showed that YTA treatment reversed the metabolic changes in colitis mice. Volcano plots exhibited that YTA could upregulate 1136 fecal metabolites and downregulate 156 fecal metabolites compared with colitis mice (Figure 5C). A total of 56 metabolites including LA were significantly altered among three groups (Figure 5D; Supplementary Figures S6I, J; Supplementary Table S6). Among these, fatty acids accounted for 25% of differential metabolites significantly altered by YTA (Figure 5E). KEGG analysis revealed that the most significant pathway affected by YTA involved LA metabolism (Figure 5F). LA occupied an important position in significantly differential metabolites (Figure 5G). Metabolites including LA, AA, docosahexaenoic acid (DHA), and 3,7,12-Trihydroxycholestan-26-al (T-hy-CT-26), which occupied key positions in KEGG and network analysis, were changed significantly (Figure 5H). LA was the only differential metabolite across the three metabolomic datasets (Supplementary Figure S7), among which fatty acids accounted for a relatively high proportion. These results suggested that LA might be an important messenger in influencing UC.

### 3.6 YTA ameliorated gut bacteria disorder in DSS-induced colitis mice

**FIGURE 6:**
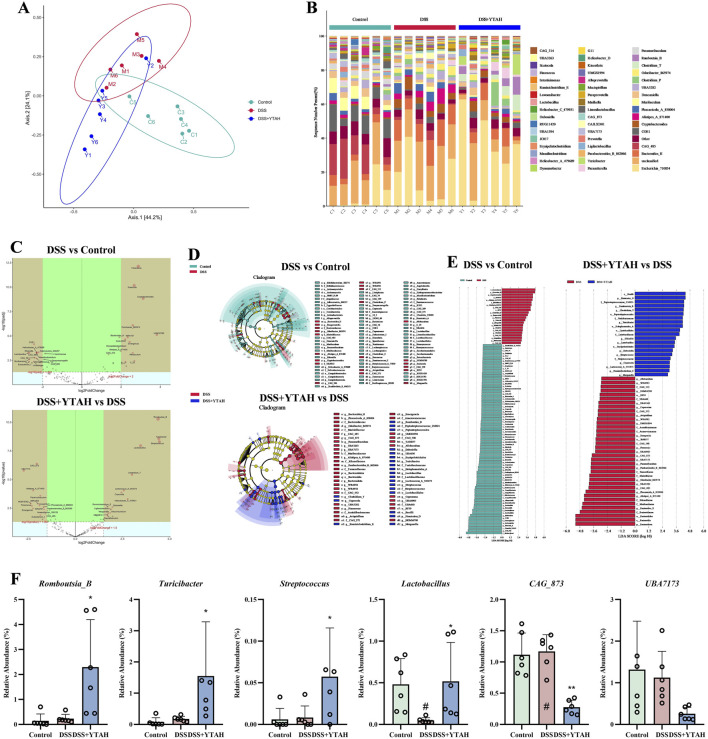
Gut microbiota diversity analysis in feces through YTA treatment on DSS-induced colitis in mice. **(A)** PCoA analysis, and **(B)** Accumulation diagram of the gut microbiota among three groups in mice feces. **(C)** DEseq2 volcano plot. **(D)** LEfse cladogram. **(E)** LDA scores in control and DSS. **(F)** Representative abundance of gut microbiota. **(A**–**F)** n = 6 per group. Mean values ±SD are presented, *P* values were calculated using One-Way ANOVA, ^##^ *P* < 0.01 compared with control, ^**^ *P* < 0.01 and ^*^ *P* < 0.05 compared with DSS.

We further explored the regulation of YTA on gut bacteria in feces from colitis mice through 16S rRNA sequencing. The Chao1 and Shannon indices indicated that YTA could increase the gut microbiota diversity of colitis mice (Supplementary Figures S8A, B). PCoA exhibited that the gut bacteria of the YTAH group shifted back from DSS group toward the control group, bringing it closer to normal (Figure 6A), and YTA treatment could restore gut bacteria diversity reduced by colitis (Figure 6B). Specifically, differential expression analysis for sequence data 2 (DESeq2) analysis and LEfSe demonstrated that YTA group had a decrease in the abundances of *Fimenecus*, *Emergencia*, and *HGM16780* compared with colitis mice, whereas these taxa were upregulated in the DSS group compared with the control group. In contrast, the abundances of *Romboutsia_B*, *Turicibacter*, *Streptococcus*, and *Lactobacillus* exhibited an increase in YTA group relative to colitis mice, while *CAG_873* and *UBA7173* exhibited a decrease in YTA group relative to colitis mice (Figures 6C–E). Combined with DESeq2 analysis and LEfSe, the abundances of differential bacteria regulated by YTA was shown in Figure 6F, which exhibited that *Romboutsia_B*, *Turicibacter*, *Streptococcus*, *Lactobacillus*, and *CAG_873* significantly alter by YTA (Figure 6F). These results suggested that YTA could restore the composition of gut microbiota.

### 3.7 Correlation analysis of gut microbiota, metabolites, and pharmacodynamic indices

**FIGURE 7:**
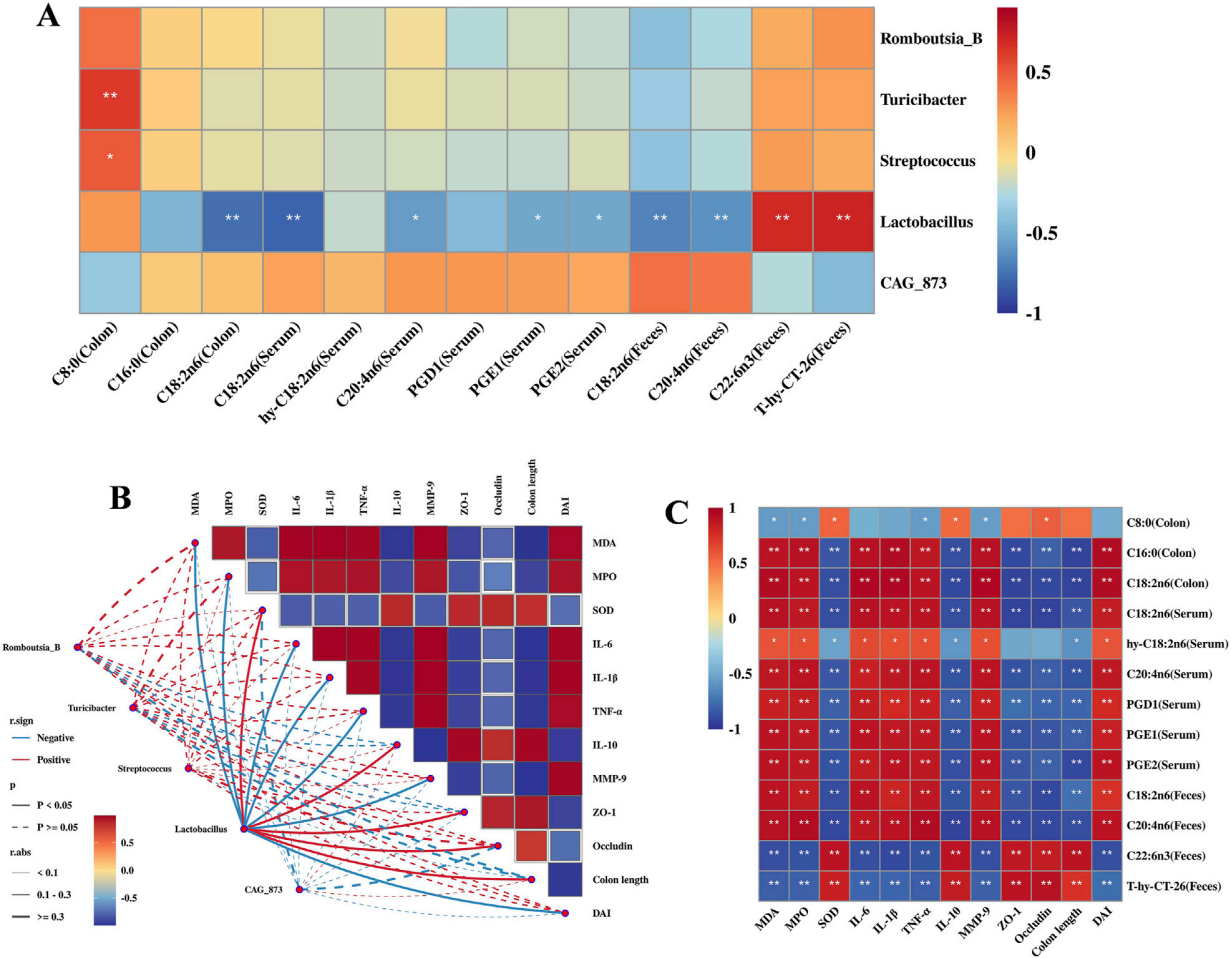
Correlation analysis. **(A)** Correlation heatmap between gut microbiota and metabolites. **(B)** Correlation network heatmap between gut microbiota and pharmacodynamic indices. **(C)** Correlation heatmap between metabolites and pharmacodynamic indices. **(A)** and **(B)** n = 6 per group, and **(C)** n = 8 per group. *P* values were calculated using Spearman’s correlation analysis, ^**^ *P* < 0.01 and ^*^ *P* < 0.05.

Based on gut bacteria, metabolites, and pharmacodynamic indices, Spearman’s correlation analysis was applied to examine their correlation. The result showed that *Lactobacillus* was significantly associated with metabolites, exhibiting a significant negative correlation with LA in colon, serum and feces, while showing a significant positive correlation with AA and T-hy-CT-26 (Figure 7A). Additionally, *Lactobacillus* was significant negatively correlated with pharmacodynamic indices. Inflammatory cytokines were significantly positively correlated with oxidative stress and negatively correlated with intestinal barrier function, while oxidative stress was significantly negatively correlated with intestinal barrier function (Figure 7B). Further correlation analysis between metabolites and pharmacodynamic indices revealed that PA, AA, LA, PGD1, PGE1, and PGE2 were significantly positively correlated with oxidative factors, inflammatory cytokines and DAI scores while significantly negatively correlated with antioxidants, anti-inflammatory cytokines, intestinal barrier function, and colon length. DHA and T-hy-CT-26 showed the opposite trends (Figure 7C). LA in colon, serum, and feces were significantly correlation with pharmacodynamic indices. The results indicated that *Lactobacillus* was closely correlated with LA, oxidative stress, inflammation, and intestinal barrier function.

### 3.8 YTA alleviated DSS-induced colitis through gut microbiota in mice

**FIGURE 8:**
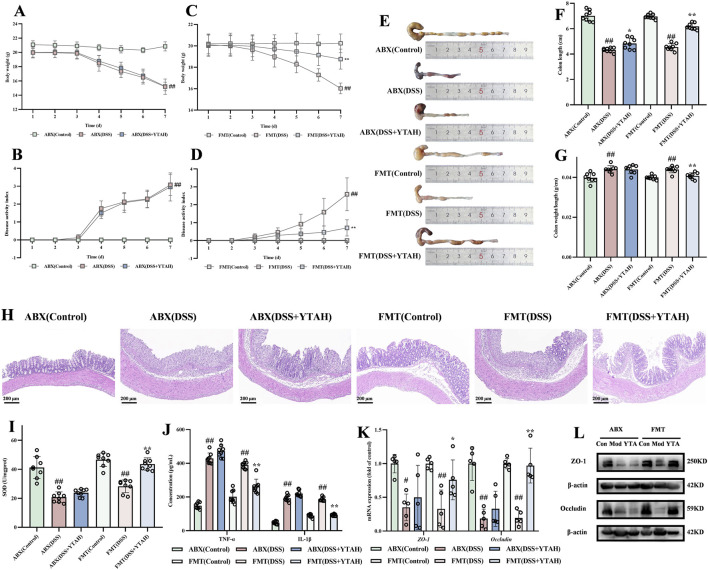
The effect of intestinal flora in DSS-induced colitis mice. **(A)** Changes of body weight, and **(B)** Changes of DAI score in ABX. **(C)** Changes of body weight, and **(D)** Changes of DAI score in FMT. **(E)** Representative morphologies of colon. **(F)** Colon length. **(G)** The ration of weight and length in colon. **(H)** H&E staining of colon tissue. **(I)** SOD, **(J)** TNF-α and IL-1β, **(K)** The mRNA levels, and (L) The protein expression of ZO-1 and occludin in colon tissue. **(A**–**G)**, **(I)** and **(J)** n = 8 per group, **(H)** and **(L)** n = 3 per group, and **(K)** n = 5 per group. Mean values ±SD are presented, *P* values were calculated using One-Way ANOVA, ^##^ *P* < 0.01 compared with control, ^**^ *P* < 0.01 and ^*^ *P* < 0.05 compared with DSS.

To validate YTA’s effectiveness in treating UC by regulating gut microbiota, we set up a PGF mouse model with an antibiotic cocktail. Body weight, DAI scores, and colon length in YTA-treated mice resembled those in the DSS group (Figures 8A,B,E–G), indicating that YTA’s effects were abolished without the presence of gut microbiota. H&E staining showed that both YTA-treated and the colitis groups exhibited similar results, such as disappearance of intestinal gland and inflammatory cell infiltration (Figure 8H). To further verify the therapeutic effect of gut microbiota modified by YTA, FMT was implemented. As shown in Figures 8C–G, FMT from YTA-treated mice to PGF colitis mice ameliorated weight loss, DAI scores, and colon shortening. These results demonstrated that recipient mice exhibited similar therapeutic effects as YTA-treated mice in colitis, indicating that YTA mediated its effect by modifying gut microbiota to treat colitis effectively. Besides, the results in PGF mice exhibited that SOD, IL-1β, and TNF-α levels were not notably altered in YTA treatment (Figures 8I,J), and the expressions of occludin and ZO-1 were also not significantly affected (Figures 8K,L), while the results in FMT mice exhibited that oxidative stress and inflammation were alleviated, and the intestinal barrier was repaired. In conclusion, YTA had no obvious effect in PGF mice, while recipient mice exhibited therapeutic effects comparable to those of YTA-treated mice, confirming that YTA exerted its therapeutic effect in colitis by modifying gut microbiota. In brief, YTA could better exert its antioxidant, anti-inflammatory, and intestinal barrier repairing effects only under the action of gut microbiota.

### 3.9 LJ was enriched by YTA and exerted anti-inflammatory activity *in vitro*

**FIGURE 9:**
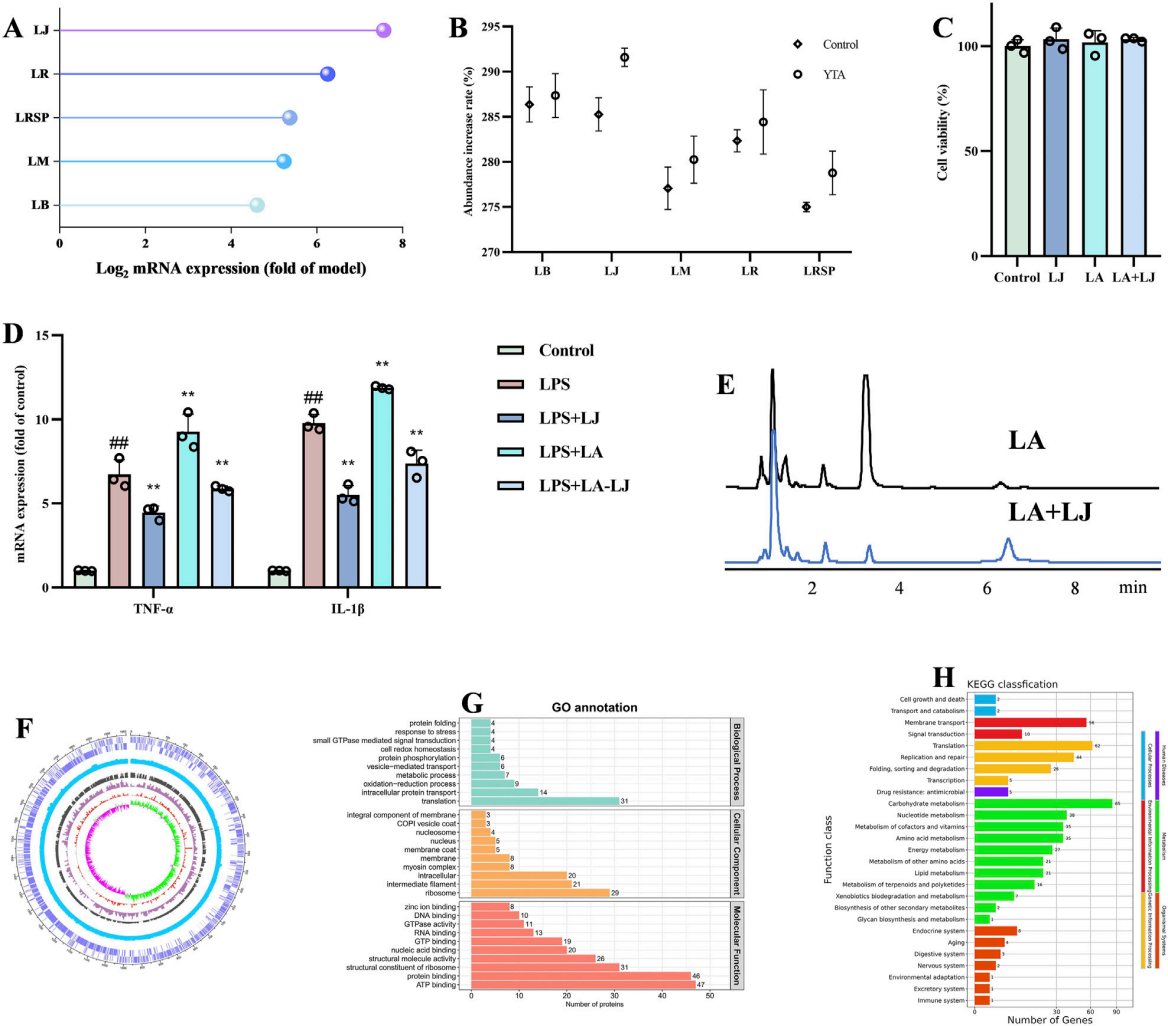
LJ regulated inflammation *in vitro*. **(A)** Changes of five *Lactobacillus* spp. in mice feces. **(B)** Relative abundance of five *Lactobacillus* spp. after incubation with YTA (presented in percent initial levels). **(C)** Cell viability, and **(D)** The mRNA levels of TNF-α and IL-1β in Caco2. **(E)** Liquid chromatography of LJ decomposing LA. **(F)** Circos, **(G)** GO classification, and **(H)** KEGG classification of LJ. **(A)** n = 5 per group, **(B**–**D)** n = 3 per group. Mean values ±SD are presented, *P* values were calculated using One-Way ANOVA, ^##^ *P* < 0.01 compared with control, ^**^ *P* < 0.01 and ^*^ *P* < 0.05 compared with LPS.

To further validate the key bacterium involved in colitis treatment, five species of *Lactobacillus* spp. were isolated from feces in mice, including *Limosilactobacillus balticus* (LB), LJ, *Ligilactobacillus murinus* (LM), *Limosilactobacillus reuteri* (LR), and *Limosilactobacillus reuteri* subsp. *Reuteri* (LRSP). We explored the abundance of these five *Lactobacillus* species in mice feces, and the results indicated that LJ was the most significantly enriched in the feces of colitis mice treated with YTA (Figure 9A). YTA could also increase the relative abundance of LJ by 1.91-fold *in vitro* (Figure 9B; Supplementary Figures S9A−E). We further explored the effect between LJ and YTA, and the MS data showed that LJ could not degrade the components of YTA but increased some metabolites. The metabolites generated by total gut microbiota included fatty acids, amino acids, and polyhydroxy compounds, and increased with culturing time (Supplementary Figures S10A−C; Supplementary Table S7).

Cellular experiments *in vitro* targeting LA were conducted to further investigate its biological role. As shown in Figures 9C,D, LA upregulated TNF-α and IL-1β expression in Caco-2 cells to aggravate the inflammatory response. When LJ was co-cultured with LA, which (Figures 9E; Supplementary Figures S11A, B) showed LJ could decompose LA within 1 h, demonstrating it could break down excessive LA to exert an anti-inflammatory effect (Figure 9D). In order to gain a better understanding of LJ’s function, we conducted a further examination of its genome. GO annotation identified 67 genes related to metabolic processes, which made up the highest proportion (Figures 9F,G). Likewise, 288 genes related to metabolism through KEGG annotation (Figure 9H), of which three genes were related to fatty acid biosynthesis and degradation. Gene-H0I41_RS06775 was preliminarily identified as a linoleate isomerase and was inferred to be involved in LA decomposition. These results suggested that LJ could be enriched by YTA both *in vitro* and *in vivo*, and possessed the ability to decompose LA to contribute anti-inflammatory activity.

### 3.10 LJ alleviated DSS-induced colitis

**FIGURE 10:**
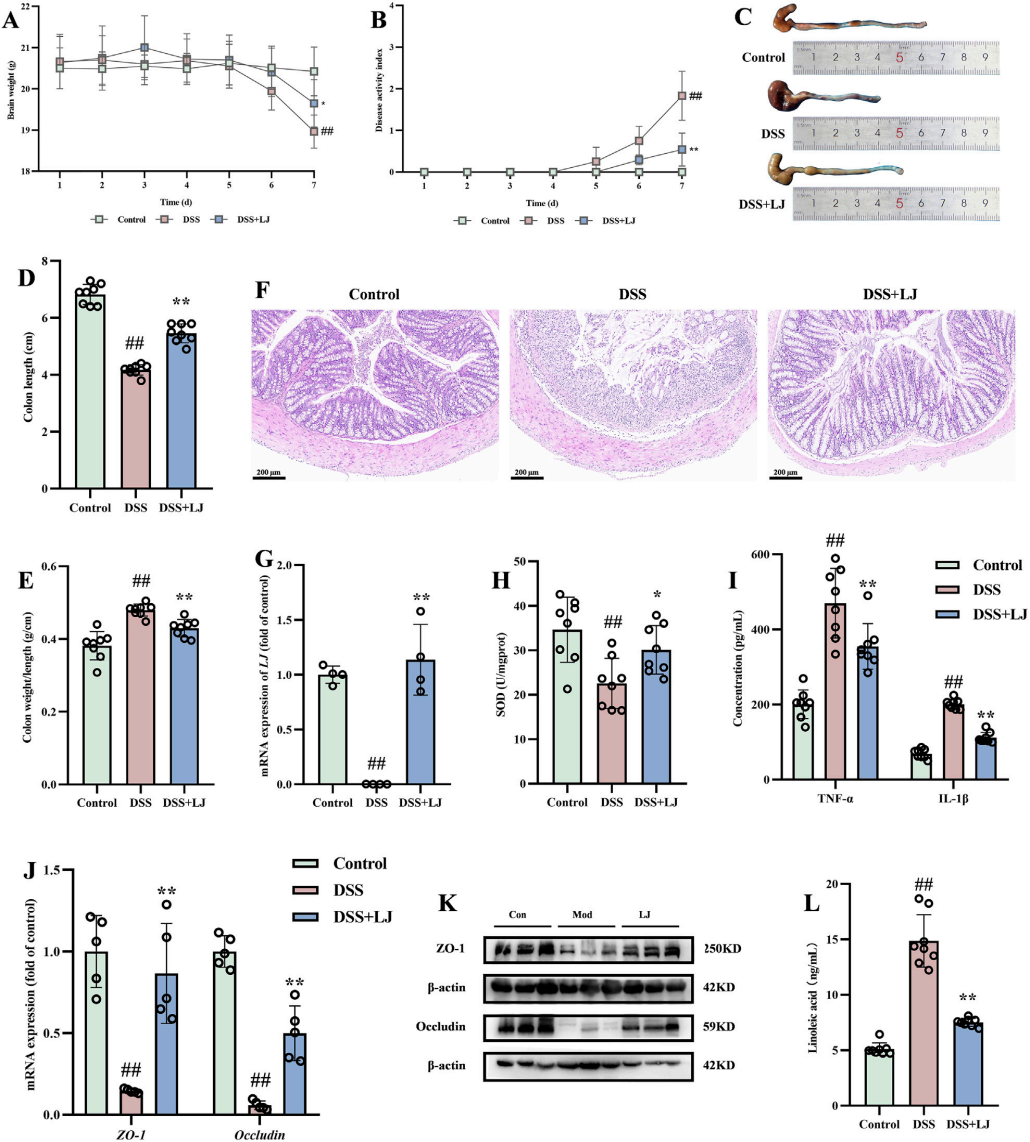
LJ regulated DSS-induced colitis in mice. **(A)** Changes of body weight. **(B)** Changes of DAI score. **(C)** Representative morphologies of colon. **(D)** Colon length. **(E)** The ration of weight and length in colon. **(F)** H&E staining of colon tissue. **(G)** The mRNA levels of LJ. **(H)** SOD, **(I)** TNF-α and IL-1β, **(J)** The mRNA levels, and **(K)** The protein expression of ZO-1 and occludin in colon tissue. **(L)** LA levels in colon tissue. **(A**–**E)**, **(H)**, **(I)**, and (L) n = 8 per group, **(F)** and **(K)** n = 3 per group, and **(G)** and **(J)** n = 5 per group. Mean values ±SD are presented, *P* values were calculated using One-Way ANOVA, ^##^ *P* < 0.01 compared with control, ^**^ *P* < 0.01 and ^*^ *P* < 0.05 compared with DSS.

Furthermore, we investigated the *in vivo* effect of LJ. We orally administered LJ to colitis mice for 7 days and evaluated its anti-colitis effect. As shown in Figures 10A–E, LJ treatment alleviated weight loss, reduced DAI scores, and mitigated colon shortening in colitis mice. LJ could also reduce the infiltration of inflammatory cells, restore the lost intestinal glands, and repair the damage to the mucosal layer (Figure 10F). Moreover, qPCR analysis showed that LJ treatment increased the relative abundance of LJ in feces (Figure 10G). Oral administration of LJ could also have effects in suppressing oxidative stress, inflammation, and restoring intestinal barrier damages (Figures 10H–K). Additionally, LJ treatment could reduce level of LA (Figure 10L). In conclusion, we found that LJ enriched by YTA could alleviate symptoms of UC, which provided a specific example for gut microbiota regulating metabolism to improve UC.

## 4 Discussion

UC represents a complex inflammatory bowel disorder characterized by persistent, relapsing, and nonspecific intestinal ulcerations, affecting approximately 4 million individuals worldwide (Le Berre et al., 2023). The therapeutic management of UC remains challenging due to the heterogeneous pathogenesis and intricate pathological progression of the disease. TCMs, extensively utilized in China and various Asian countries, may offer an alternative therapeutic approach for UC patients. YHL has been conventionally used in *Zhong Hua Ben Cao* to alleviate various UC symptoms, including diarrhea, hematochezia, and abdominal pain. These symptoms correspond to UC in modern clinical manifestations (Zhao et al., 2021), which is attributed to the large intestine meridian corresponding to the location of intestinal diseases. Nevertheless, the precise mechanisms underlying the therapeutic efficacy of YTA against UC remain to be fully elucidated. Our study determined that YTA could ameliorate UC through metabolomics profiling and composition of gut bacteria in reducing inflammation, oxidative stress as well as restoring the intestinal barrier. We identified and isolated LJ, which was associated with differential metabolites, could also ameliorated UC. These results aligned with the traditional indications of YHL.

In our study, the significantly differential metabolites in colon, serum, and fecal metabolomics were lipids. Notably, among these lipids, LA was identified as a differential metabolite in colon, serum, and fecal metabolomics, and consistent results confirmed that LA played a central role in the therapeutic effect of YTA on colitis mice, with other metabolites associated with LA. LA plays a pivotal role in the pathogenesis of UC through multiple mechanisms. It can induce the production of inflammatory metabolites that stimulate the occurrence of UC, such as 13(S)-HODE (Duan et al., 2023) and 13-OxoODE (Warner et al., 2021). LA also reduces endocannabinoids, which are crucial for preventing inflammation; their depletion thereby exacerbates the inflammatory response. It alters the composition of gut bacteria, including increasing the levels of *E. coli* and reducing the death of beneficial microbes like *Lactobacillus*. Additionally, LA can damage the intestinal barrier by binding to HNF-4α, leading to intestinal leakage and further elevating the risk of inflammation and colitis (Zhang et al., 2020). We further explored whether YTA could modify other metabolites associated with LA to exert its therapeutic effect, based on the central role of LA in UC pathogenesis. The results showed that YTA could modify other metabolites related to LA, such as AA in serum and feces, PA in colon, DHA in feces, and CA in colon, which AA promotes inflammation, DHA inhibits inflammation, and CA acts synergistically with LA in metabolic regulation. AA is synthesized from LA through a series of metabolic reactions. AA induces inflammation by increasing the production of inflammatory substances via cyclooxygenase (COX), lipoxygenase, and cytochrome P450, which generate prostaglandins, leukotrienes, and epoxyeicosatrienoic acids, respectively (Wang et al., 2021). Prostaglandins in serum could be significantly reduced by YTA, and they also occupied a key position in serum metabolomics. PA competes with LA for enzymes such as acyl-CoA synthetase, involved in absorption and metabolism (Pope and Dixon, 2023). PA induces inflammation by activating inflammatory signaling pathways such as nuclear factor-κB (NF-κB), NOD-like receptor thermal protein domain associated protein 3 (NLRP3), and pyruvate kinase isozyme typeM2 (PKM2) (Fatima et al., 2019). DHA is related to LA in that they share the same metabolic enzymes, with which they have competitions and balances. DHA exerts anti-inflammatory effects, through increasing anti-inflammatory substances (e.g., resolvin D1 and protectin D1), regulating transcription factors such as NF-κB and peroxisome proliferator-activated receptors (PPARs), and regulating the activity of immune cells such as macrophages and neutrophils (Serhan, 2014). CA can cooperate with LA in the process of metabolic regulation, thereby inhibiting the release of inflammatory cytokines and the growth of harmful bacteria (Yang et al., 2023). YTA could also regulate bile acids and other lipids. Bile acids promote the lipids digestion and absorption by emulsifying fats and activating lipase, and indirectly affect the expression of genes related to fatty acid metabolism by activating signaling pathways such as the farnesol X receptor (FXR) (Cai et al., 2022). In summary, LA directly promoted inflammation through multiple pathways such as inducing inflammatory metabolites and damaging the intestinal barrier. And the imbalance of its related metabolites including AA, PA, DHA, and CA further amplified this effect. Therefore, metabolic abnormalities, particularly LA, were precisely the key link that triggered the interaction between inflammation and oxidative stress and drove the progression of UC, and YTA could modify the metabolites network correlated with LA to treat UC.

The changes in metabolites can lead to alterations in conditions such as inflammation and oxidative stress, and these two factors can also trigger each other, and cause damage to intestinal epithelial cells, exacerbating UC lesions (Tian et al., 2024). YTA had the advantage of being multi-component, and thus treated UC through multiple functions including anti-inflammation, antioxidation and barrier repair compared with monomers such as berberine, palmatine, and sanguinarine (Li et al., 2016; Jing et al., 2021; Ji et al., 2024; Niu et al., 2013). In our study, we discovered that YTA could remarkably reduce pro-inflammatory cytokines levels including TNF-α, IL-1β, and IL-6, while promoting anti-inflammatory cytokines IL-10 level. Since these cytokines are primarily secreted by macrophages, we further assessed the expression of MMP-9, a key inflammatory mediator associated with macrophage activation. The results showed that MMP-9 levels were markedly decreased following YTA treatment, revealing that YTA could exert anti-inflammatory effects by modulating macrophage-mediated immune responses. YTA could also significantly reduce levels of oxidants such as MPO and MDA, while increasing the activity of antioxidant enzyme SOD. YTA could inhibit inflammation and oxidative stress which suggested that the vicious cycle between inflammation and oxidative stress may be broken by YTA. Our study found that oxidative stress were associated with inflammation. Previous studies also have confirmed that immune cells such as macrophages activated under inflammatory conditions can produce a large amount of reactive oxygen species (ROS) through respiratory burst, and the accumulation of ROS will further activate inflammatory signaling pathways such as NF-κB, promoting the release of cytokines such as TNF-α and IL-1β, and forming a feedback loop (Song J. et al., 2023). In this study, YTA reduced pro-inflammatory cytokines to limit ROS production from immune cells and enhanced antioxidant capacity to clear excess ROS. And this dual action confirmed that YTA could break the vicious cycle as initially hypothesized. When inflammation and ROS attack the intestine, tight junction proteins in the colon (e.g., ZO-1 and occludin) are downregulated, leading to damage to intestinal barrier function and decreased ability to resist external invaders (Zhu et al., 2024). Our study exhibited that YTA restored intestinal barrier function, consistent with previous reports. Inflammation can lead to erosion and ulceration of the intestinal mucosa, prompting excessive secretion of intestinal fluid, impairing colonic absorption function, and ultimately causing diarrhea and weight loss (Caliendo et al., 2023). Our findings demonstrated that YTA could inhibit diarrhea and mitigate weight loss, thereby improving the UC symptoms. Inflammatory stimulation and fibrous tissue growth can lead to a shorter colon and increase ratio of colon weight and length, while YTA could reverse them. Therefore, as a multi-component agent, YTA could improve the colonic physiological state by alleviating inflammation, reducing oxidative stress, and restoring the intestinal barrier, thereby ameliorating UC pathology.

Trillions of intestinal microbes maintain a dynamic balance in the body and are intimately linked to metabolic processes, including LA metabolism. Alterations in their composition and function may drive disease onset or progression, and also have a significant impact on the LA metabolism (de Vos et al., 2022; Sommer et al., 2017). Conversely, abnormalities in LA metabolism may also have a feedback effect on the structure and physiological roles of gut bacteria. In the context of UC, this bidirectional balance of gut microbiota and metabolites is disrupted, and YTA may act by restoring this balance. The diversity and abundance of gut microbiota decrease in colitis patients (Walujkar et al., 2018), and YTA could restore this situation, which indicated YTA could regulate the gut microbiota to treat UC. Research has shown that, at the phylum level, the abundances of *Firmicutes* and Bacteroidetes are decreased whereas *Proteobacteria* is increased in UC (Andoh, 2016). Similar results were observed in our study, and we further found that YTA could regulate the abundance of *Firmicutes*. Moreover, YTA could significantly increase the abundance of *Romboutsia_B*, *Turicibacter*, *Streptococcus*, and *Lactobacillus*. Among them, *Romboutsia* can convert primary bile acids into secondary bile acids to regulate glyceryl phosphatide metabolism, thereby achieving antioxidative and anti-inflammatory effects (Ferrara et al., 2021; Wei et al., 2025). *Turicibacter* can modify host bile acids through different bile salt hydrolase to change lipids in serum, thereby restoring the disrupted metabolism (Lynch et al., 2023). *Streptococcus* can affect lipid metabolism and regulate the intestinal immune system by participating in the conversion of bile acids including reabsorption and excretion of bile acids and stimulating the production of beneficial metabolites (Brown et al., 2023; Jing et al., 2025). *Lactobacillus* can regulate the balance of gut microbiota by secreting antibacterial substances to inhibit harmful bacteria. It can also activate the phagocytic function of macrophages, thereby stimulating the intestinal mucosal immune system to produce immunoglobulin A and regulating immune responses. Additionally, it can regulate unsaturated fatty acids, affect lipid absorption, and produce anti-inflammatory metabolites like butyric acid to modulate inflammatory signaling pathways (Mejia-Caballero and Marco, 2025). Conversely, YTA could reduce *CAG_873* and *UBA7173*. Among them, *CAG_873* can increase inflammatory metabolites to promote the release of inflammatory cytokines (Shen et al., 2025), while *UBA7173* can penetrate the mucus layer, damage the intestinal mucosal barrier, and thereby trigger inflammation (Kordahi et al., 2025). The findings indicated that imbalance of gut bacteria was at least one of the causes of UC. A decrease in beneficial bacteria might exacerbate UC. *Lactobacillus* showed significant changes among three groups, with a marked decline observed in colitis mice compared with control and a significant rebound in YTA treatment group. The correlation heat map showed that *Lactobacillus* was significantly related to LA, DHA, and T-hy-CT-26, indicating that *Lactobacillus* could interact with LA, DHA, and T-hy-CT-26 to exert effects, with the strongest correlation with LA among them. Reports have shown that *Lactobacillus* can convert LA to conjugated LA via linoleate isomerase to reduce inflammation (Song X. et al., 2023), it can also enhance the absorption efficiency of DHA by optimizing the intestinal environment and improving the barrier function of the intestinal mucosa (Fang et al., 2024), and it can help maintain the balance of the bile acid pool and reduce the abnormal accumulation of bile acids in the liver and intestines (Gu et al., 2025). Additionally, *Lactobacillus* was significantly correlated with pharmacodynamic indices, which demonstrated that *Lactobacillus* could regulate metabolites to exert antioxidant, anti-inflammatory, and intestinal barrier restoring effects. To directly confirm whether gut microbiota are required for YTA’s therapeutic effects, we conducted ABX and FMT experiments. Findings from the ABX experiment clearly showed that YTA almost lost its antioxidant and anti-inflammatory effects when gut microbiota was eliminated, while YTA restored the important therapeutic effects when gut microbiota was transplanted. These results mutually confirmed that the therapeutic effects of YTA largely depended on the synergistic action of the gut microbiota.

We found that *Lactobacillus* was not only a significantly beneficial biomarker, but also played an important role in preventing and treating diarrhea in clinical practice as reported (Gutierrez-Castrellon et al., 2014). Therefore, we isolated five *Lactobacillus* species from mice feces, and we found that LJ could be significantly enriched by YTA *in vitro* and *in vivo*. Moreover, some metabolites increased when LJ was cultured with YTA *in vitro* such as monoamines, pyrimidines and aromatic compounds, which could promote the growth of LJ by enhancing metabolic adaptability and providing growth factors (Mayers et al., 2024; Zhang et al., 2025). Oral administration of LJ to colitis mice could reduce symptoms of UC and inhibit inflammation and oxidative stress. Additionally, qPCR results exhibited that LJ abundance was dramatically increased in colitis mice treated by YTA, indicating that LJ could colonize in the intestinal tract. Gut bacteria can affect host metabolites to influence disease status. Previous reports have shown that accumulation of LA could induce macrophage infiltration and proinflammatory cytokine expression in macrophages to increase colitis susceptibility (Zhang et al., 2024). Consistent with the ability of YTA to regulate LA, we found that LJ could also decompose LA, and genomic analysis suggested that the most likely enzyme was Gene-H0I41_RS06775 which was annotated as linoleate isomerase. Collectively, LJ emerged as a central mediator of YTA’s anti-colitic effects, which not only decomposed excess LA *via* linoleate isomerase to inhibit inflammation, but also directly suppressed inflammation, reduced oxidative stress, and upregulated tight junction proteins. The results linked gut microbiota modulation and metabolic balance, and explained that the therapeutic benefits of YTA depended on the presence of gut microbiota, with LJ playing a key role.

## 5 Conclusion

Our study demonstrates that YTA ameliorates UC by reducing inflammatory cytokines and oxidative stress, restoring intestinal barrier, regulating the metabolic network, and rebuilding the gut microbiota. YTA regulates abnormal metabolic profiles in colon, serum, and feces, most prominently by reducing LA levels. Gut microbiota depletion and FMT experiments show that therapeutic effects of YTA largely depend on the synergistic action of the gut microbiota. LJ, isolated from mice feces, is enriched both *in vitro* and *in vivo*, and ameliorates UC by reducing inflammatory cytokines and oxidative stress, as well as restoring intestinal barrier. Collectively, these findings highlight YTA as a prospective therapeutic candidate for UC.

## Data Availability

The sequencing data has been deposited at https://ngdc.cncb.ac.cn/, accession number CRA019815. Further inquiries can be directed to the corresponding authors.
