# Supplementary material for: Total alkaloids of Corydalis saxicola Bunting ameliorate ulcerative colitis through regulation of metabolite networks and gut microbiota
Source: Front Pharmacol. 2025 Dec 16;16:1721116. doi: 10.3389/fphar.2025.1721116 (PMC12748159; doi:10.3389/fphar.2025.1721116)
Supplement: Supplementary file 1 [file Supplementaryfile1.docx]

**Supplementary Materials**

**Method S1.**

An appropriate amount of YHL was extracted with 78% ethanol (pH = 2) for 1.9 h at 90°C. The crude extract of total alkaloids was then dissolved in 0.2% hydrochloric acid. The solution was adsorbed onto XDA-8 macroporous resin and followed by elution with pure water and 90% ethanol. The ethanol eluent was collected and subjected to vacuum concentration until it dried. Added 2 mg dried power to 50 mL methanol, then take the supernatant and pass it through a 0.22um filter membrane. Weighted appropriate allocryptopine (b20109, Yuanye), epiberberine (b20108, Yuanye), columbamine (b20391, Yuanye), jatrorrhizine (b21476, Yuanye), dehydrocavidine (b50084, Yuanye), sanguinarine (b21412, Yuanye), berberine (b21379, Yuanye), palmatine (m100844, Meryer), 1-hydroxypalmatine (self-made), and chelerythrine (b20052, Yuanye) to methanol, then take the supernatant and pass it through a 0.22um filter membrane. The analysis was jointly an HPLC and an ESI mass spectrometer (Agilent Technologies Inc., Santa Clara, CA, USA). The mobile phases were aqueous with 0.1% formic acid (A) and acetonitrile-methanol (1:1, v/v) (B). The gradient elution conditions were as follows: 0-10 min, 17-21% B; 10-15 min, 21-21% B; 15-25 min, 21-22% B; 25-30 min, 22-22% B; 30-40 min, 22-35% B; 40-45 min, 35-40% B; 45-70 min, 40-90% B. The chromatographic column used a C18 column (4.6 × 250 mm, 5 μm, Welch Sci & Tech, Shanghai, China), with column temperature maintained at 35°C and a flow rate set at 1 mL/min. Collision energy set at 0, 20, 40 psi at positive and negative modes. Masshunter 6.0 and Microsoft Excel were used to analyze results.

**Method S2.**

Metabolites in serum and colon were extracted using a methanol/acetonitrile/aqueous solution (2:2:1, v/v). The analysis was performed using an Agilent 1290 Infinity LC in combination with a TripleTOF 6600 mass spectrometer (AB Sciex, Foster City, CA, USA). The mobile phases consisted of 2mM sodium acetate and 2mM ammonium solution (A) and acetonitrile (B). The elution gradient was: 0-1.5 min, 98%-98% B; 1.5-12 min, 98%-2% B; 12-14 min, 2%-2% B; 14-14.1 min, 2%-98% B; 14.1-17 min, 98%-98% B. The injection volume was 2 μL, and the chromatographic column was an ACQUITY UPLC BEH Amide column (2.1 × 100 mm, 1.7 μm, Waters, Massachusetts, USA), with a thermostat set to 25°C and a flow rate of 0.3 mL/min. Data were processed using XCMS and Microsoft Excel, and results were visualized using Simca 14.1 software.

Metabolites in feces were extracted using methanol and analyzed with an SCIEX Exion LC in combination with a TripleTOF 5600 mass spectrometer (AB Sciex, Foster City, CA, USA). The mobile phases were 0.1% aqueous formic acid (A) and acetonitrile (B). The elution gradient was: 0-5 min, 3%-8% B; 5-11 min, 8%-30% B; 11-20 min, 30%-80% B; 20-21 min, 80%-95% B; 21-27 min, 95%-95% B; 27-32 min, 95%-3% B. The injection volume was 2 μL, and the chromatographic column used was a C18 column (4.6 × 250 mm, 5 μm, Welch Sci & Tech, Shanghai, China), with a thermostat set to 35°C and a flow rate of 0.3 mL/min. Mass spectrometric data were processed using Markerview 1.3.0 and Microsoft Excel.

**Method S3.**

The mobile phases used were methanol/acetonitrile/aqueous solution (1:1:1, v/v/v) containing 2mM ammonium acetate (A) and isopropanol/acetonitrile (1:1, v/v) with 2mM ammonium acetate (B). The elution gradient was: 0-1 min, 20%-20% B; 1-6 min, 20%-80% B; 6-10 min, 80%-95% B; 10-12 min, 95%-95% B. A C18 chromatographic column (2.1 × 100 mm, 5 μm, Welch Sci & Tech, Shanghai, China) was applied, the column thermostat maintained at 30°C and a flow rate set at 0.3 mL/min. Collision energy set at 0, 20, 40 psi at positive and negative modes. Masshunter 6.0 and Microsoft Excel were used to analyze results.

Table S1. Primer sequences.

| **Primer** | **Sequence** |
| --- | --- |
| *β-actin*-F | GGCTGTATTCCCCTCCATCG |
| *β-actin*-R | CCAGTTGGTAACAA TGCCATGT |
| *Occludin*-F | ATGTCCGGCCGATGCTCTC |
| *Occludin*-R | TTTGGCTGCTCTTGGGTCTGTAT |
| *ZO-1*-F | CTTCTCTTGCTGGCCCTAAAC |
| *ZO-1*-R | TGGCTTCACTTGAGGTTTCTG |
| *Total bacteria*-F | ACTCCTACGGGAGGCAGCAG |
| *Total bacteria*-R | ATTACCGCGGCTGCTGG |
| *LB-F* | ACCTGATTGACGATGGATC |
| *LB-R* | AAATGTTATCCCCCGCTC |
| *LJ-F* | TGCGGTGCATTAGCTAGTTG |
| *LJ-R* | ATTCCCTACTGCTGCCTC |
| *LM-F* | ACCCGAACTGAGAGGTTG |
| *LM-R* | ATCCGAAGACCTTCTTCACC |
| *LR-F* | ACGATGGATCACCAGTG |
| *LR-R* | AACCATGCGGCTTTTG |
| *LRSP-T* | ACATGCAAGTCGTACGCACTG |
| *LRSP-F* | GATCCATCGTCAATCAGGTGCAAG |
| *β-actin*-human-F | TCCTTCCTGGGCATGGAGT |
| *β-actin*-human-R | AGCACTGTGTTGGCGTACAG |
| *TNF-α*-human-F | TGCTTGTTCCTCAGCCTCTTCTCC |
| *TNF-α*-human-R | AAGTGCAGCAGGCAGAAGAGC |
| *IL-1β*-human-F | ATGAAGTGCTCCTTCCAGG |
| *IL-1β*-human-R | AGCCCTTGCTGTAGTGG |

Table S2. Antibodies used for western blotting analysis.

| **Antibodies** | **Dilution** | **Source** | **Cat#** |
| --- | --- | --- | --- |
| Occludin | 1:1000 | Proteintech | 27260-1-AP |
| ZO-1 | 1:1000 | Huabio | HA722797 |
| β-Actin | 1:20000 | Proteintech | 66009-1-lg |
| HRP-conjugated anti-rabbit lgG | 1:4000 | Proteintech | SA00001-7L |
| HRP-conjugated anti-mouse lgG | 1:2000 | Proteintech | SA00001-1 |

Table S3. Characterization of alkaloids in YTA by HPLC–DAD-Q-TOF-MS/MS.

| **No** | **tR (min)** | **UV (nm)** | **Quasimolecular (p) [M+/M+ H/Na/NH_4_]^+^ (Error, ppm)** | **Molecular formula** | **Molecular formula generator** | **MS/MS fragments (p)** | **Proposed compound** |
| --- | --- | --- | --- | --- | --- | --- | --- |
| A1 | 4.553 | 280, 370 | 314.1753 (-0.54) | C_19_H_23_NO_3_ | 313.1678 | 298, 269, 237, 175, 165, 107 | Lotusine |
| A2 | 6.373 | 228, 275 | 316.1541 (2.85) | C_18_H_21_NO_4_ | 315.1471 | 178, 177, 163, 137 | N-methyl-3'-O-methylcoclaurine |
| A3 | 6.806 | 225, 265, 305(sh) | 342.1718 (-4.25) | C_20_H_24_NO_4_ | 342.1705 | 298, 297, 282, 266, 265, 237, 222, 207, 191, 179 | Magnoflorine |
| A4 | 7.846 | 229, 258, 295, 330 | 300.1593 (0.61) | C_18_H_21_NO_3_ | 299.1521 | 192, 177, 165, 107 | N-methylcoclaurine |
| A5 | 11.486 | 232, 280(sh), 350 | 342.1699 (-0.14) | C_20_H_24_NO_4_ | 342.1705 | 310, 192, 177, 165, 150, 131, 118 | Cyclanoline |
| A6 | 13.739 | 236, 282, 330 | 342.1701 (-0.93) | C_20_H_24_NO_4_ | 342.1705 | 326, 294, 267, 240, 192, 178, 163, 151, 134 | Tetrahydrocolumbamine |
| A7 | 14.606 | 232, 290(sh), 320 | 298.1449 (-3.00) | C_18_H_19_NO_3_ | 297.1365 | 192, 176, 159, 148, 131, 121, 107 | Cinnamolaurine |
| A8 | 15.646 | 242(sh), 270, 355 | 322.1082 (-2.03) | C_19_H_16_NO_4_ | 322.1079 | 322, 307, 294, 279, 266, 251, 250 | Tetradehydrocheilanthifoline |
| A9 | 16.513 | 238(sh), 270, 342 | 324.1236 (-1.31) | C_19_H_18_NO_4_ | 324.1236 | 309, 294, 282, 280, 266, 255, 238 | Stepharanine |
| A10 | 16.946 | 246, 276(sh), 340 | 354.1331 (1.79) | C_20_H_19_NO_5_ | 353.1263 | 355, 338, 324, 310, 296, 278, 266, 262, 236, 206, 196, 188, 178, 160 | Protopine |
| A11 | 18.159 | 234, 280, 340 | 314.1755 (-0.48) | C_19_H_23_NO_3_ | 313.1678 | 192, 177, 176, 121, 106 | N-demethylcolletine |
| A12 | 18.419 | 230 280 | 324.1229 (0.99) | C_19_H_18_NO_4_ | 324.1236 | 309, 294, 281, 280, 266, 251, 238 | Dehydrodiscretamine |
| A13 | 19.719 | 240, 280, 344 | 368.1498 (-0.81) | C_21_H_22_NO_5_ | 368.1498 | 352, 338, 324, 310, 292, 280, 262, 234, 149 | 13-Hydroxypalmatine |
| A14 | 20.499 | 240(sh), 276, 342 | 324.1239 (-2.08) | C_19_H_18_NO_4_ | 324.1236 | 309, 294, 281, 280, 266, 252, 238 | Demethyleneberberine |
| A15 | 21.279 | 234, 282, 332 | 370.1663 (-2.82) | C_21_H_23_NO_5_ | 369.1576 | 370, 352, 336, 290, 206, 189, 188, 181, 179, 165 | Allocryptopine |
| A16 | 21.973 | 232, 275(sh), 350 | 336.1224 (2.06) | C_20_H_18_NO_4_ | 336.1236 | 336, 321, 306, 293, 276, 264 | Dehydroapocavidine |
| A17 | 26.666 | 250, 310, 365 | 312.1612 (-4.27) | C_19_H_21_NO_3_ | 311.1521 | 312, 121 | Pronuciferine |
| A18 | 23.186 | 240(sh)270, 345 | 336.1234 (-0.59) | C_20_H_18_NO_4_ | 336.1236 | 336, 322, 321, 306, 293, 276, 264, | Dehydroisoapocavidine |
| A19 | 24.226 | 242(sh), 270, 350 | 320.0928 (-3.05) | C_19_H_14_NO_4_ | 320.0923 | 320, 318, 293, 292, 291, 277, 262, 249, 234, 220, 205 | Coptisine |
| A20 | 26.306 | 244(sh), 270, 350 | 336.1234 (-0.82) | C_20_H_18_NO_4_ | 336.1236 | 336, 320, 308, 292, 278, 275, 262, 249 | Epiberberine |
| A21 | 28.126 | 240(sh), 275, 340 | 338.1401 (-3.28) | C_20_H_20_NO_4_ | 338.1392 | 338, 308, 294, 279, 265, 251, 237 | Columbamine |
| A22 | 27.866 | 245(sh), 272, 342 | 354.1704 (-0.78) | C_21_H_23_NO_4_ | 353.1627 | 308, 248, 192, 165, 150, 133, 105 | Thalictrifoline |
| A23 | 29.253 | 236(sh), 270, 340 | 338.1399 (-3.10) | C_20_H_20_NO_4_ | 338.1392 | 338, 323, 322, 308, 307, 294, 279, 262 | Jatrorrhizine |
| A24 | 32.459 | 245(sh), 276, 336 | 354.1339 (-0.90) | C_20_H_19_NO_5_ | 353.1263 | 355, 339, 324, 306, 293, 278, 266, 250, 238, 222, 208, 192 | Papaveraldine |
| A25 | 34.366 | 241(sh), 268, 345 | 334.1077 (-3.45) | C_20_H_16_NO_4_ | 334.1079 | 334, 319, 306, 291, 276, 261, 248, 233, 220, 205, 191, 147 | Corysamine |
| A26 | 35.319 | 240, 270, 345 | 350.1391 (-4.91) | C_21_H_20_NO_4_ | 350.1392 | 350, 336, 335, 334, 306 | Dehydrocavidine |
| A27 | 37.659 | 240(sh)265, 340 | 336.1233 (-3.75) | C_20_H_18_NO_4_ | 336.1236 | 336, 321, 320, 306, 304, 292, 278 | Berberine |
| A28 | 38.266 | 245(sh), 270, 340 | 352.1517 (-4.06) | C_21_H_22_NO_4_ | 352.1549 | 352, 337, 336, 322, 308, 294, 278 | Palmatine |
| A29 | 39.913 | 245(sh), 278, 340 | 368.1504 (-1.91) | C_21_H_22_NO_5_ | 368.1498 | 369, 352, 338, 324, 310, 307, 295, 292, 277, 263, 249, 234 | 1-Hydroxypalmatine |
| A30 | 42.252 | 268, 318, 340(sh) | 348.1246 (-3.92) | C_21_H_18_NO_4_ | 348.1236 | 348, 332, 318, 304, 290, 275, 260, 246, 232, 217 | Chelerythrine |

Table S4. Differential metabolites in colon.

| **No** | **m/z** | **Mode** | **Metabolites** | **HMDB** | **DSS&Control** | | | | **DSS+YTAH&DSS** | | | |
| --- | --- | --- | --- | --- | --- | --- | --- | --- | --- | --- | --- | --- |
|  |  |  |  |  | **VIP** | ***P*(corr)** | **Log_2_FC** | ***P*** | **VIP** | ***P*(corr)** | **Log_2_FC** | ***P*** |
| 1 | 165.9791 | NEG | aAmid | HMDB0000064 | 1.0737 | 0.6332 | 1.4895 | 0.0116 | 1.1693 | -0.4715 | -0.9742 | 0.0494 |
| 2 | 854.5690 | POS | PC(18:1/18:1) | HMDB0000593 | 1.7238 | 0.5701 | 0.9931 | 0.0174 | 2.4524 | -0.5362 | -0.9098 | 0.0332 |
| 3 | 856.5831 | POS | PC(18:1/22:5) | HMDB0008088 | 2.0504 | 0.6769 | 1.2541 | 0.0026 | 2.3426 | -0.5779 | -0.8004 | 0.0218 |
| 4 | 742.5742 | POS | PC(14:0/20:1) | HMDB0007879 | 1.6138 | -0.6054 | -0.8232 | 0.0180 | 1.9297 | 0.5883 | 0.6711 | 0.0181 |
| 5 | 325.2171 | NEG | C18:2n6 | HMDB0000673 | 2.0848 | 0.7768 | 1.6023 | 0.0001 | 2.0970 | -0.5789 | -0.7792 | 0.0171 |
| 6 | 189.0670 | NEG | C8:0 | HMDB0000482 | 2.2736 | -0.6400 | -1.2428 | 0.0044 | 2.9234 | 0.6374 | 1.0880 | 0.0053 |
| 7 | 317.2123 | NEG | LTA4 | HMDB0001337 | 3.9903 | 0.6995 | 1.5348 | 0.0023 | 4.1662 | -0.5281 | -0.8454 | 0.0392 |
| 8 | 353.2334 | NEG | PGD1 | HMDB0005102 | 5.9536 | 0.7141 | 2.1146 | 0.0021 | 7.7352 | -0.6605 | -1.6917 | 0.0055 |
| 9 | 421.2728 | NEG | hy-CT | HMDB0000717 | 1.7710 | -0.7968 | -1.2494 | 0.0002 | 1.5003 | 0.5379 | 0.7414 | 0.0312 |
| 10 | 361.2386 | NEG | hy-C22:5n6 | HMDB0010214 | 1.9977 | 0.6004 | 1.4394 | 0.0090 | 2.4363 | -0.5383 | -1.0794 | 0.0334 |
| 11 | 431.2570 | NEG | hy-C20:5n3 | HMDB0010202 | 1.6641 | -0.7812 | -1.3365 | 0.0003 | 1.4636 | 0.6519 | 0.7017 | 0.0095 |
| 12 | 300.0394 | NEG | C16:0 | HMDB0000220 | 1.5958 | 0.9047 | 4.5029 | 0.0000 | 1.3561 | -0.6128 | -0.8046 | 0.0123 |
| 13 | 239.1654 | NEG | C10:0 | HMDB0000511 | 2.8704 | 0.8401 | 1.9866 | 0.0001 | 2.5786 | -0.5965 | -0.7718 | 0.0111 |
| 14 | 355.2494 | NEG | hy-C20:4n5 | HMDB0006111 | 2.2693 | 0.6928 | 1.6252 | 0.0024 | 2.3944 | -0.5221 | -0.8693 | 0.0453 |
| 15 | 351.2177 | NEG | PGE2 | HMDB0001220 | 3.4380 | 0.6354 | 0.9267 | 0.0066 | 3.8542 | -0.5134 | -0.6436 | 0.0427 |
| 16 | 765.5247 | POS | PC(14:1/18:4) | HMDB0007910 | 1.0222 | 0.5234 | 0.7390 | 0.0292 | 1.6495 | -0.5597 | -0.7093 | 0.0258 |
| 17 | 447.2519 | NEG | C22:3n3 | HMDB0002823 | 3.0195 | -0.4520 | -1.7181 | 0.0697 | 2.9339 | 0.6707 | 0.8220 | 0.0064 |
| 18 | 215.1653 | NEG | hy-C12:0 | HMDB0002059 | 1.1832 | 0.7319 | 1.0013 | 0.0011 | 1.4461 | -0.6576 | -0.7112 | 0.0077 |
| 19 | 724.5277 | POS | PE(22:2/14:0) | HMDB0009547 | 1.5906 | -0.5128 | -0.9064 | 0.0405 | 2.5937 | 0.5510 | 0.9193 | 0.0294 |
| 20 | 716.5586 | POS | PC(16:0/16:0) | HMDB0000564 | 2.2374 | -0.4995 | -1.1244 | 0.0406 | 2.1797 | 0.4812 | 0.8372 | 0.0485 |
| 21 | 449.2663 | POS | CPA(18:2/0:0) | HMDB0007007 | 1.6357 | -0.7850 | -1.7938 | 0.0002 | 1.1759 | 0.5190 | 0.9209 | 0.0445 |
| 22 | 140.9863 | NEG | hy-C3:0 | HMDB0000139 | 2.5655 | -0.5578 | -0.8824 | 0.0132 | 4.2121 | 0.5538 | 1.0004 | 0.0315 |
| 23 | 764.5591 | POS | PC(22:4/14:0) | HMDB0008462 | 2.8174 | -0.9273 | -1.4383 | 0.0000 | 1.7323 | 0.6333 | 0.5292 | 0.0069 |
| 24 | 738.5436 | POS | PC(20:3/14:0) | HMDB0008394 | 1.3613 | -0.7214 | -0.9514 | 0.0012 | 1.2106 | 0.5547 | 0.5340 | 0.0217 |
| 25 | 520.2583 | NEG | LysoPE(0:0/18:3) | HMDB0011478 | 1.0878 | -0.6259 | -1.1121 | 0.0062 | 1.0789 | 0.5781 | 0.7909 | 0.0155 |
| 26 | 518.3620 | NEG | LysoPE(22:0/0:0) | HMDB0011520 | 1.9286 | -0.8462 | -1.7069 | 0.0000 | 1.0600 | 0.5546 | 0.5475 | 0.0234 |
| 27 | 452.3134 | POS | LysoPA(19:0/0:0) | HMDB0114746 | 1.7970 | -0.6136 | -0.7747 | 0.0062 | 1.9227 | 0.4855 | 0.5099 | 0.0468 |
| 28 | 149.9947 | NEG | Ser | HMDB0003406 | 1.0416 | 0.7194 | 1.3616 | 0.0011 | 1.1628 | -0.5834 | -0.7900 | 0.0230 |
| 29 | 462.2995 | NEG | LysoPE(0:0/18:0) | HMDB0011129 | 2.0636 | -0.7783 | -1.2696 | 0.0003 | 1.4919 | 0.5514 | 0.5982 | 0.0358 |
| 30 | 141.0168 | NEG | cb-C1:0 | HMDB0000119 | 4.1725 | 0.8002 | 1.1850 | 0.0003 | 5.5645 | -0.6915 | -1.0351 | 0.0038 |
| 31 | 485.2220 | NEG | MG(22:4/0:0/0:0) | HMDB0011584 | 1.1894 | -0.6394 | -1.9568 | 0.0098 | 1.4352 | 0.5846 | 1.5597 | 0.0176 |
| 32 | 487.2375 | NEG | CA | HMDB0000619 | 2.4668 | -0.5135 | -1.4074 | 0.0514 | 3.8155 | 0.5611 | 1.3767 | 0.0238 |
| 33 | 87.0090 | NEG | C4:0 | HMDB0000039 | 1.1991 | -0.6695 | -0.6476 | 0.0035 | 1.7710 | 0.5074 | 0.7622 | 0.0388 |
| 34 | 104.0707 | POS | CE | HMDB0000097 | 3.2152 | -0.5956 | -1.0583 | 0.0145 | 3.5753 | 0.5189 | 0.8217 | 0.0326 |
| 35 | 136.0616 | POS | Glyd | HMDB0000043 | 1.9943 | 0.7309 | 0.9655 | 0.0022 | 2.3929 | -0.6245 | -0.7821 | 0.0101 |

Table S5. Differential metabolites in serum.

| **No** | **m/z** | **Mode** | **Metabolites** | **HMDB** | **DSS&Control** | | | | **DSS+YTAH&DSS** | | | |
| --- | --- | --- | --- | --- | --- | --- | --- | --- | --- | --- | --- | --- |
|  |  |  |  |  | **VIP** | ***P*(corr)** | **Log_2_FC** | ***P*** | **VIP** | ***P*(corr)** | **Log_2_FC** | ***P*** |
| 1 | 172.9925 | NEG | ca-C4:0 | HMDB0000193 | 14.2869 | -0.5331 | -1.0437 | 1.6664 | 25.0698 | 0.5645 | 0.0342 | 0.0146 |
| 2 | 227.9974 | NEG | PLP | HMDB0001491 | 1.4483 | -0.6463 | -0.9095 | 0.9762 | 2.0392 | 0.7344 | 0.0077 | 0.0009 |
| 3 | 393.2779 | NEG | MG(0:0/18:0/0:0) | HMDB0011535 | 1.1144 | -0.5674 | -0.6595 | 0.7298 | 1.4373 | 0.6767 | 0.0234 | 0.0048 |
| 4 | 432.2217 | NEG | LysoPC(10:0/0:0) | HMDB0003752 | 1.9215 | -0.9271 | -1.8435 | 0.9974 | 1.3113 | 0.7449 | 0.0000 | 0.0005 |
| 5 | 655.4730 | NEG | DG(20:5/18:3/0:0) | HMDB0007568 | 1.1003 | 0.7868 | 0.9122 | -0.7173 | 1.1770 | -0.6854 | 0.0003 | 0.0028 |
| 6 | 325.2174 | NEG | C18:2n6 | HMDB0000673 | 2.1374 | 0.7003 | 1.6795 | -1.1455 | 2.2291 | -0.5708 | 0.0037 | 0.0272 |
| 7 | 860.4812 | NEG | PC(18:3/18:1) | HMDB0008202 | 1.3433 | -0.7063 | -0.7769 | 0.5079 | 1.2081 | 0.5098 | 0.0034 | 0.0453 |
| 8 | 736.5195 | NEG | PE(18:0/14:0) | HMDB0008986 | 10.9963 | 0.7380 | 0.9154 | -1.1026 | 14.8950 | -0.7496 | 0.0010 | 0.0008 |
| 9 | 321.2438 | NEG | hy-C20:4n6 | HMDB0062747 | 2.3947 | 0.8682 | 1.4225 | -0.7223 | 2.3018 | -0.8254 | 0.0000 | 0.0001 |
| 10 | 317.2123 | NEG | ox-C20:4n6 | HMDB0013633 | 6.1852 | 0.9307 | 1.7459 | -0.9339 | 6.2560 | -0.8744 | 0.0000 | 0.0000 |
| 11 | 295.2279 | NEG | po-C18:2n6 | HMDB0004701 | 4.6422 | 0.6229 | 0.9955 | -0.5948 | 4.3602 | -0.5014 | 0.0092 | 0.0461 |
| 12 | 629.5154 | NEG | TG(18:0/8:0/8:0) | HMDB0107807 | 1.6567 | 0.7650 | 0.9006 | -0.7679 | 1.7332 | -0.6044 | 0.0011 | 0.0097 |
| 13 | 202.0510 | NEG | ThDP | HMDB0060484 | 1.7105 | -0.8120 | -2.0072 | 1.2859 | 1.4798 | 0.7770 | 0.0002 | 0.0008 |
| 14 | 429.3013 | NEG | MG(24:6/0:0/0:0) | HMDB0011590 | 1.7838 | -0.8730 | -2.1362 | 1.0821 | 1.1865 | 0.6816 | 0.0000 | 0.0064 |
| 15 | 820.5831 | POS | PE(18:1/22:2) | HMDB0009041 | 2.7136 | 0.8675 | 1.8814 | 0.0000 | 2.4280 | -0.6075 | -0.8333 | 0.0132 |
| 16 | 520.2473 | NEG | LysoPE(20:5/0:0) | HMDB0011519 | 1.4411 | 0.7740 | 2.1850 | -0.8066 | 1.1166 | -0.5044 | 0.0009 | 0.0396 |
| 17 | 337.2384 | NEG | C24:6n3 | HMDB0002007 | 1.2885 | 0.7414 | 1.4691 | -0.9163 | 1.2906 | -0.6225 | 0.0015 | 0.0139 |
| 18 | 335.2230 | NEG | PGE1 | HMDB0001442 | 2.4952 | 0.7672 | 2.6970 | -1.7200 | 2.7553 | -0.7032 | 0.0007 | 0.0035 |
| 19 | 472.2472 | NEG | LysoPE(18:4/0:0) | HMDB0011510 | 1.0647 | 0.8860 | 2.8104 | -1.3657 | 1.0562 | -0.7389 | 0.0000 | 0.0012 |
| 20 | 241.0831 | NEG | His-Ser | HMDB0000273 | 7.8077 | 0.9192 | 0.8552 | -0.5572 | 7.5784 | -0.7644 | 0.0000 | 0.0004 |
| 21 | 299.2593 | NEG | CT-C15 | HMDB0001551 | 1.3013 | -0.6855 | -0.6121 | 0.6693 | 1.8267 | 0.7402 | 0.0020 | 0.0012 |
| 22 | 377.2335 | NEG | po-C20:4n2 | HMDB0341284 | 1.4778 | 0.7633 | 2.7288 | -1.0037 | 1.3626 | -0.6077 | 0.0003 | 0.0159 |
| 23 | 758.5693 | POS | DG(20:1/22:6/0:0) | HMDB0007411 | 28.3105 | 0.7718 | 0.9170 | 0.0004 | 28.4068 | -0.5785 | -0.5057 | 0.0145 |
| 24 | 101.0244 | NEG | ox-C4:0 | HMDB0000005 | 2.4415 | 0.8216 | 1.4092 | -0.7136 | 2.2223 | -0.6811 | 0.0001 | 0.0050 |
| 25 | 771.5742 | NEG | TG(15:0/14:0/14:1) | HMDB0042966 | 1.0794 | 0.5471 | 0.6799 | -0.6493 | 1.2832 | -0.5050 | 0.0346 | 0.0414 |
| 26 | 741.5310 | NEG | DG(22:6/22:2/0:0) | HMDB0007784 | 1.2915 | 0.8114 | 0.7437 | -0.6208 | 1.4531 | -0.7311 | 0.0002 | 0.0015 |
| 27 | 786.6005 | POS | PC(18:1/18:1) | HMDB0000593 | 12.8493 | 0.7739 | 1.1125 | 0.0006 | 12.4518 | -0.6405 | -0.5618 | 0.0092 |
| 28 | 113.0245 | NEG | cb-hy-C5:0 | HMDB0002545 | 7.7664 | -0.8617 | -1.2474 | 0.8884 | 7.6360 | 0.8104 | 0.0000 | 0.0003 |
| 29 | 509.2891 | NEG | LysoPG(18:1/0:0) | HMDB0240602 | 1.6124 | 0.9527 | 1.5809 | -0.9412 | 1.6990 | -0.9194 | 0.0000 | 0.0000 |
| 30 | 507.2733 | NEG | LysoPG(18:2/0:0) | HMDB0240600 | 2.2990 | 0.9244 | 1.8886 | -0.7677 | 1.9872 | -0.7715 | 0.0000 | 0.0005 |
| 31 | 243.0624 | NEG | Urd | HMDB0000296 | 1.5321 | -0.6124 | -0.6085 | 0.0149 | 1.9018 | 0.5617 | 0.5835 | 0.0250 |
| 32 | 448.3423 | POS | ct-C18:1n9 | HMDB0005065 | 1.2067 | 0.6082 | 0.6878 | 0.0132 | 1.6445 | -0.6387 | -0.5798 | 0.0135 |
| 33 | 429.3771 | POS | CTA | HMDB0003822 | 1.3104 | 0.7866 | 1.5127 | 0.0003 | 1.4896 | -0.6895 | -0.8995 | 0.0041 |
| 34 | 424.3422 | POS | ct-C18:2n6 | HMDB0006469 | 4.2171 | 0.7514 | 1.0575 | 0.0010 | 5.4202 | -0.7190 | -0.8741 | 0.0020 |
| 35 | 400.3422 | POS | ct-C16:0 | HMDB0000222 | 3.5789 | 0.6495 | 0.7724 | 0.0061 | 4.3960 | -0.5742 | -0.5958 | 0.0229 |
| 36 | 129.0659 | POS | hy-Thy | HMDB0000079 | 1.7581 | -0.7223 | -1.1355 | 0.0018 | 1.5899 | 0.6531 | 0.6539 | 0.0079 |
| 37 | 396.3110 | POS | hy-C18:2n6 | HMDB0003871 | 2.0244 | 0.6557 | 1.3535 | 0.0051 | 2.6007 | -0.6102 | -1.0230 | 0.0136 |
| 38 | 370.2952 | POS | C20:3n9 | HMDB0010378 | 2.0580 | 0.6334 | 0.7659 | 0.0072 | 2.9448 | -0.6675 | -0.7239 | 0.0055 |
| 39 | 368.2795 | POS | Glyd | HMDB0005096 | 1.4245 | 0.6694 | 0.7641 | 0.0042 | 1.7901 | -0.6369 | -0.6017 | 0.0101 |
| 40 | 556.2977 | NEG | LysoPC(18:1/0:0) | HMDB0002815 | 2.5602 | 0.5745 | 0.8063 | -0.9535 | 3.2581 | -0.6618 | 0.0206 | 0.0059 |
| 41 | 568.3929 | POS | LysoPE(0:0/20:1) | HMDB0011482 | 1.8108 | 0.9040 | 1.0525 | 0.0000 | 2.5372 | -0.9079 | -1.0575 | 0.0000 |
| 42 | 385.2738 | POS | PGE2 | HMDB0001220 | 1.4398 | 0.8942 | 1.2963 | 0.0000 | 1.4869 | -0.7659 | -0.6540 | 0.0007 |
| 43 | 568.3398 | POS | LysoPC(18:0/0:0) | HMDB0010384 | 13.3702 | 0.8939 | 0.7616 | 0.0000 | 15.8303 | -0.8280 | -0.5337 | 0.0001 |
| 44 | 552.3098 | NEG | LysoPE(24:6/0:0) | HMDB0011529 | 6.2944 | 0.8761 | 1.0306 | -0.9028 | 7.4555 | -0.8945 | 0.0000 | 0.0000 |
| 45 | 504.3098 | NEG | LysoPE(0:0/20:2) | HMDB0011483 | 6.5665 | 0.7190 | 0.5149 | -0.6372 | 9.2689 | -0.7941 | 0.0033 | 0.0003 |
| 46 | 524.2786 | NEG | LysoPE(22:6/0:0) | HMDB0011526 | 10.2639 | 0.9218 | 1.4058 | -0.6543 | 9.4292 | -0.8458 | 0.0000 | 0.0000 |
| 47 | 337.2738 | POS | C20:4n6 | HMDB0001043 | 1.5868 | 0.7715 | 1.1593 | 0.0003 | 1.9250 | -0.7023 | -0.8333 | 0.0019 |
| 48 | 476.2786 | NEG | LysoPE(18:2/0:0) | HMDB0011507 | 6.5438 | 0.9278 | 1.3355 | -0.6856 | 6.1596 | -0.8154 | 0.0000 | 0.0002 |
| 49 | 177.0405 | NEG | GA | HMDB0000150 | 1.5662 | -0.7087 | -1.7423 | 1.0679 | 1.3760 | 0.6686 | 0.0010 | 0.0075 |
| 50 | 412.3059 | POS | PC(16:0/20:4) | HMDB0007982 | 1.3709 | 0.7237 | 1.6013 | 0.0013 | 1.6873 | -0.6517 | -1.1101 | 0.0068 |
| 51 | 386.2903 | POS | ct-C18:3n6 | HMDB0006318 | 1.7356 | 0.7561 | 1.3931 | 0.0006 | 2.0687 | -0.6794 | -0.9389 | 0.0042 |
| 52 | 384.2746 | POS | hy-C20:4n5 | HMDB0006111 | 1.4208 | 0.7623 | 1.2981 | 0.0005 | 1.6932 | -0.7030 | -0.8743 | 0.0027 |
| 53 | 361.2738 | POS | PGD1 | HMDB0005102 | 2.0585 | 0.8413 | 2.0180 | 0.0000 | 2.6388 | -0.8167 | -1.6381 | 0.0002 |
| 54 | 619.2892 | NEG | CAG | HMDB0002577 | 6.0796 | 0.8530 | 2.1597 | -1.7043 | 7.1040 | -0.8250 | 0.0000 | 0.0001 |
| 55 | 643.2863 | POS | LysoPI(20:4/0:0) | HMDB0061690 | 1.1309 | 0.8764 | 1.8579 | 0.0000 | 1.4436 | -0.8395 | -1.4424 | 0.0001 |
| 56 | 595.2893 | NEG | LysoPI(18:2/0:0) | HMDB0240597 | 1.7021 | 0.9206 | 1.7704 | -1.5085 | 2.0234 | -0.9016 | 0.0000 | 0.0000 |
| 57 | 347.1503 | POS | C20:5n3 | HMDB0001999 | 1.4338 | -0.7700 | -1.9944 | 0.0009 | 1.8039 | 0.7012 | 1.8115 | 0.0037 |
| 58 | 190.0975 | POS | Hom | HMDB0000679 | 5.5010 | -0.7095 | -1.3802 | 0.0031 | 7.2964 | 0.6640 | 1.3104 | 0.0070 |
| 59 | 132.0808 | POS | Asp | HMDB0000168 | 2.2256 | 0.6687 | 5.5715 | 0.0080 | 3.1711 | -0.6887 | -6.3855 | 0.0076 |
| 60 | 189.0667 | NEG | Ala-Thr | HMDB0028697 | 2.0687 | -0.6923 | -1.3039 | 0.0048 | 2.9559 | 0.7068 | 1.4740 | 0.0025 |
| 61 | 144.1018 | POS | Prod | HMDB0004827 | 3.0238 | -0.5355 | -1.1890 | 0.0284 | 5.9133 | 0.5888 | 1.6829 | 0.0205 |
| 62 | 124.0074 | NEG | Tau | HMDB0000251 | 13.7105 | -0.9413 | -0.8777 | 0.5708 | 12.9959 | 0.8970 | 0.0000 | 0.0000 |
| 63 | 101.0244 | NEG | cb-me-C2:0 | HMDB0000060 | 1.9166 | -0.5579 | -1.5753 | 0.5866 | 1.4250 | 0.7976 | 0.0273 | 0.0003 |
| 64 | 179.0557 | NEG | Hex-2-ulose | HMDB0001266 | 3.2708 | -0.8211 | -1.7379 | 0.0002 | 3.1527 | 0.8354 | 1.2413 | 0.0001 |
| 65 | 180.0670 | NEG | Tyrd | HMDB0000866 | 3.0922 | -0.8912 | -1.7904 | 0.7861 | 1.7743 | 0.5126 | 0.0000 | 0.0449 |
| 66 | 195.0508 | NEG | GDL | HMDB0000625 | 1.7849 | -0.8565 | -1.2106 | 0.8413 | 1.7618 | 0.8157 | 0.0000 | 0.0003 |
| 67 | 105.0194 | NEG | hy-C3:0 | HMDB0031818 | 1.2208 | -0.8184 | -1.0784 | 1.0033 | 1.4542 | 0.7598 | 0.0001 | 0.0005 |
| 68 | 117.0662 | POS | hy-C5 | HMDB0000508 | 6.5999 | -0.8234 | -1.1842 | 0.0001 | 6.0088 | 0.7740 | 0.6479 | 0.0007 |
| 69 | 173.0932 | NEG | Ornd | HMDB0003357 | 1.8520 | -0.6562 | -0.8393 | 0.7148 | 2.1137 | 0.6902 | 0.0071 | 0.0032 |
| 70 | 195.0505 | NEG | hy-GDL | HMDB0003466 | 3.2345 | -0.8120 | -1.4734 | 0.8401 | 2.5375 | 0.5484 | 0.0003 | 0.0277 |

Table S6. Differential metabolites in feces.

| **No** | **m/z** | **Mode** | **Metabolites** | **HMDB** | **DSS&Control** | | | | **DSS+YTAH&DSS** | | | |
| --- | --- | --- | --- | --- | --- | --- | --- | --- | --- | --- | --- | --- |
|  |  |  |  |  | **VIP** | ***P*(corr)** | **Log_2_FC** | ***P*** | **VIP** | ***P*(corr)** | **Log_2_FC** | ***P*** |
| 1 | 131.0698 | NEG | hy-me-C5:0 | HMDB0000317 | 1.1523 | 0.5892 | 1.3232 | 0.0156 | 1.5671 | -0.7298 | -2.1245 | 0.0018 |
| 2 | 167.0197 | NEG | Asp | HMDB0000168 | 1.8184 | 0.8459 | 2.5602 | 0.0000 | 2.0728 | -0.8842 | -4.6941 | 0.0000 |
| 3 | 201.1107 | NEG | ca-C9:0 | HMDB0059719 | 1.1082 | -0.9070 | -3.7529 | 0.0000 | 1.6269 | 0.9490 | 4.6251 | 0.0000 |
| 4 | 206.0407 | NEG | Cysd | HMDB0030411 | 1.7823 | -0.7416 | -3.3240 | 0.0014 | 3.4780 | 0.9605 | 4.7228 | 0.0000 |
| 5 | 218.1003 | NEG | VB5 | HMDB0000210 | 1.3913 | -0.8585 | -1.1560 | 0.0000 | 1.4154 | 0.6315 | 1.2660 | 0.0125 |
| 6 | 229.1518 | NEG | Val-Leu | HMDB0029131 | 1.5922 | -0.7969 | -1.4915 | 0.0003 | 2.1358 | 0.7923 | 2.0644 | 0.0003 |
| 7 | 243.1196 | NEG | Leu-Hyd | HMDB0028930 | 1.6616 | -0.9610 | -4.7922 | 0.0000 | 1.9245 | 0.9790 | 5.0589 | 0.0000 |
| 8 | 279.2298 | NEG | C18:2n6 | HMDB0000673 | 3.3180 | 0.5298 | 2.0669 | 0.0331 | 3.3832 | -0.5251 | -1.9597 | 0.0360 |
| 9 | 287.1807 | NEG | ca-hy-C14:0 | HMDB0031885 | 1.4035 | -0.9637 | -4.3819 | 0.0000 | 1.7529 | 0.9926 | 4.8468 | 0.0000 |
| 10 | 297.2762 | NEG | me-C18:0 | HMDB0340364 | 1.6346 | -0.4855 | -1.3237 | 0.0490 | 3.5738 | 0.8496 | 2.6886 | 0.0000 |
| 11 | 311.2202 | NEG | ca-C17:0 | HMDB0302976 | 1.2852 | -0.8493 | -2.6536 | 0.0001 | 1.4578 | 0.8020 | 2.9277 | 0.0003 |
| 12 | 357.2767 | POS | C22:3n7 | HMDB0031099 | 8.4460 | 0.8136 | 1.4028 | 0.0001 | 7.8948 | -0.7515 | -1.0133 | 0.0011 |
| 13 | 391.2784 | NEG | D-hy-CA | HMDB0002585 | 1.7160 | -0.9370 | -3.7538 | 0.0000 | 1.2597 | 0.9259 | 2.8649 | 0.0000 |
| 14 | 407.2786 | NEG | MCA | HMDB0000506 | 2.1954 | 0.5069 | 1.3611 | 0.0462 | 2.4485 | -0.5806 | -1.8419 | 0.0181 |
| 15 | 436.2801 | NEG | LysoPE(16:0/0:0) | HMDB0011152 | 1.4431 | -0.5895 | -1.0677 | 0.0176 | 1.2036 | -0.5502 | -1.3102 | 0.0325 |
| 16 | 471.2377 | NEG | CDCAS | HMDB0002586 | 1.5387 | -0.7013 | -3.5530 | 0.0020 | 1.1167 | 0.8594 | 2.4634 | 0.0000 |
| 17 | 476.2715 | NEG | LysoPE(18:2/0:0) | HMDB0011507 | 1.3296 | -0.8630 | -1.6495 | 0.0000 | 2.2091 | 0.8402 | 2.6744 | 0.0001 |
| 18 | 476.2771 | NEG | LysoPE(0:0/18:2) | HMDB0011477 | 2.0208 | -0.9079 | -2.7644 | 0.0000 | 1.8178 | 0.8291 | 2.4428 | 0.0001 |
| 19 | 524.2772 | NEG | LysoPE(22:6/0:0) | HMDB0011526 | 1.0187 | 0.5593 | 3.3056 | 0.0289 | 1.0199 | -0.5136 | -2.2489 | 0.0514 |
| 20 | 540.3280 | NEG | LysoPE(18:0/0:0) | HMDB0011130 | 1.1097 | 0.5503 | 1.5729 | 0.0311 | 1.3187 | -0.6049 | -1.9738 | 0.0167 |
| 21 | 588.3269 | NEG | LysoPE(0:0/22:4) | HMDB0011493 | 1.4703 | -0.8001 | -1.4200 | 0.0003 | 1.3174 | 0.6296 | 1.2376 | 0.0086 |
| 22 | 655.4739 | NEG | PA(16:1/18:2) | HMDB0011155 | 2.4534 | 0.8145 | 2.2542 | 0.0001 | 2.3690 | -0.7684 | -1.6034 | 0.0007 |
| 23 | 677.4528 | NEG | hy-PA(18:1/14:0) | HMDB0267574 | 1.3785 | 0.8933 | 2.0920 | 0.0000 | 1.2356 | -0.8230 | -1.2421 | 0.0001 |
| 24 | 781.5498 | NEG | hy-SM(18:2/20:3) | HMDB0290577 | 3.1234 | -0.8647 | -7.4906 | 0.0000 | 1.4502 | 0.8615 | 5.2019 | 0.0000 |
| 25 | 783.5646 | NEG | hy-SM(18:1/20:3) | HMDB0290525 | 6.1573 | -0.9502 | -6.4501 | 0.0000 | 3.1017 | 0.8231 | 4.5690 | 0.0001 |
| 26 | 303.2295 | NEG | C20:4n6 | HMDB0001043 | 5.8906 | 0.7899 | 0.6251 | 0.0002 | 5.6285 | -0.7393 | -0.4973 | 0.0012 |
| 27 | 229.1518 | POS | Pro-Ile | HMDB0304810 | 1.6661 | -0.7925 | 0.0003 | -1.4915 | 2.1842 | 0.7870 | 0.0003 | 2.0644 |
| 28 | 258.2035 | POS | Glyd | HMDB0013272 | 1.1217 | -0.9019 | -4.1270 | 0.0000 | 1.1680 | 0.8946 | 4.1679 | 0.0000 |
| 29 | 297.2762 | POS | C19:1n9 | HMDB0340925 | 1.6391 | -0.4795 | -1.3237 | 0.0490 | 3.6389 | 0.8518 | 2.6886 | 0.0000 |
| 30 | 355.2608 | POS | C20:1n13 | HMDB0035159 | 1.1410 | -0.6780 | -1.3637 | 0.0038 | 1.0805 | 0.6291 | 1.2522 | 0.0088 |
| 31 | 357.2758 | POS | Glud | HMDB0242050 | 5.3649 | -0.9688 | -2.5561 | 0.0000 | 3.8832 | 0.8605 | 1.8905 | 0.0000 |
| 32 | 327.2309 | NEG | C22:6n3 | HMDB0002183 | 1.5683 | -0.8518 | -2.1364 | 0.0000 | 1.0706 | 0.6878 | 1.4718 | 0.0036 |
| 33 | 360.3071 | POS | ca-C19:0 | HMDB0242141 | 5.9519 | -0.9689 | -6.2149 | 0.0000 | 2.8608 | 0.9510 | 4.1692 | 0.0000 |
| 34 | 373.2702 | POS | Lys-Ala-Arg | HMDB0341172 | 1.9381 | -0.6719 | -1.4957 | 0.0043 | 1.7392 | 0.5869 | 1.2732 | 0.0162 |
| 35 | 375.2902 | POS | M-hy-CA | HMDB0000308 | 1.8135 | -0.9681 | -2.5683 | 0.0000 | 1.3182 | 0.8616 | 1.9082 | 0.0000 |
| 36 | 379.2918 | POS | Lysd | HMDB0242055 | 2.4037 | -0.8299 | -2.0797 | 0.0001 | 4.0872 | 0.9585 | 3.2354 | 0.0000 |
| 37 | 381.3074 | POS | hy-ea-C20:4n6 | HMDB0013630 | 1.2892 | -0.9197 | -2.7599 | 0.0000 | 1.8600 | 0.9693 | 3.6384 | 0.0000 |
| 38 | 408.3103 | POS | ct-C15:0 | HMDB0062517 | 2.6694 | -0.8268 | -3.5491 | 0.0001 | 2.1631 | 0.8424 | 2.9322 | 0.0001 |
| 39 | 419.2660 | POS | CPA(18:1/0:0) | HMDB0007006 | 1.2030 | 0.5659 | 1.7652 | 0.0211 | 1.2308 | -0.5872 | -1.9056 | 0.0166 |
| 40 | 427.3521 | POS | D-hy-CT | HMDB0006893 | 1.0300 | -0.5180 | -1.8351 | 0.0356 | 3.0544 | 0.8764 | 4.2545 | 0.0000 |
| 41 | 452.3075 | POS | Phed | HMDB0062336 | 1.4425 | -0.9441 | -3.3124 | 0.0000 | 1.6502 | 0.9597 | 3.6154 | 0.0000 |
| 42 | 457.3263 | POS | T-hy-CT-27 | HMDB0062208 | 2.4340 | -0.9544 | -5.0114 | 0.0000 | 1.1908 | 0.8826 | 3.1182 | 0.0000 |
| 43 | 457.3269 | POS | T-hy-CA | HMDB0000359 | 2.1704 | -0.9417 | -5.8747 | 0.0000 | 1.1865 | 0.8539 | 4.2746 | 0.0000 |
| 44 | 457.3274 | POS | T-hy-CT-26 | HMDB0003533 | 1.4423 | -0.9102 | -3.6396 | 0.0000 | 1.4795 | 0.8488 | 3.7167 | 0.0000 |
| 45 | 473.3244 | POS | T-hy-CT | HMDB0060137 | 2.5854 | -0.8872 | -5.1120 | 0.0000 | 1.5186 | 0.9122 | 3.5824 | 0.0000 |
| 46 | 480.3049 | POS | LysoPE(18:1/0:0) | HMDB0011505 | 1.1562 | -0.7428 | -1.4788 | 0.0008 | 2.7373 | 0.9564 | 3.2800 | 0.0000 |
| 47 | 501.3169 | POS | P-hy-CA | HMDB0038495 | 2.6054 | -0.9260 | -7.0494 | 0.0000 | 1.1570 | 0.8067 | 4.7955 | 0.0002 |
| 48 | 544.3357 | POS | LysoPC(0:0/18:1) | HMDB0061701 | 3.0608 | -0.8568 | -2.3486 | 0.0000 | 2.5239 | 0.7409 | 2.0229 | 0.0010 |
| 49 | 546.3521 | POS | LysoPC(20:3/0:0) | HMDB0010394 | 1.1155 | -0.7682 | -2.1072 | 0.0005 | 1.2343 | 0.6748 | 2.3938 | 0.0041 |
| 50 | 617.4707 | POS | DG(14:1/20:5/0:0) | HMDB0007056 | 1.7521 | -0.9282 | -3.7277 | 0.0000 | 1.9356 | 0.9153 | 3.9590 | 0.0000 |
| 51 | 785.5876 | POS | DG(21:0/0:0/21:0) | HMDB0094368 | 7.7633 | -0.9590 | -6.5882 | 0.0000 | 3.8530 | 0.8318 | 4.7356 | 0.0001 |
| 52 | 807.5787 | POS | PA(18:1/24:1) | HMDB0114920 | 1.5944 | -0.9715 | -4.7095 | 0.0000 | 1.1284 | 0.8822 | 3.8279 | 0.0000 |
| 53 | 834.6057 | POS | PC(18:1/22:5) | HMDB0008089 | 4.6379 | 0.5010 | 3.0359 | 0.0483 | 4.8565 | -0.5427 | -5.2025 | 0.0300 |
| 54 | 836.6172 | POS | PC(22:1/18:4) | HMDB0008568 | 1.1344 | 0.4987 | 3.1600 | 0.0493 | 1.1825 | -0.5361 | -5.1782 | 0.0326 |
| 55 | 839.5620 | POS | PI(18:0/16:0) | HMDB0009805 | 1.1795 | 0.5161 | 3.4129 | 0.0405 | 1.2122 | -0.5409 | -4.7105 | 0.0307 |
| 56 | 994.5181 | POS | CDP-DG(18:0/20:4) | HMDB0006982 | 1.3096 | -0.5601 | -2.0916 | 0.0236 | 1.3237 | 0.5125 | 1.9301 | 0.0420 |

Table S7. Characterization of chemical constituents in YTA cultured with *Lactobacillus johnsonii* by HPLC-Q-TOF-MS/MS.

| **No** | **tR (time)** | **UV (nm)** | **Quasimolecular**  **(n)**  **[M−H]¯/ [M+Cl/COOH]¯**  **(Error, ppm)** | **Quasimolecular**  **(p) [M+/M+ H/Na/NH_4_]^+^ (Error, ppm)** | **Molecular formula** | **Molecular formula generator** | **MS/MS fragments (n)** | **MS/MS fragments (p)** | **Proposed compound** | **Source** |
| --- | --- | --- | --- | --- | --- | --- | --- | --- | --- | --- |
| 1 | 1.775 | 205 |  | 203.2217 | C_10_H_26_N_4_ | 202.2157 (4.35) |  | 129, 112, 84, 72 | Spermine | LJ48, LJ24 |
| 2 | 2.012 | 208 |  | 103.1226 | C_5_H_14_N_2_ | 102.1157 (3.10) |  | 86, 69 | Cadaverine | LJ48, LJ24 |
| 3 | 2.386 | 270 | 497.1731 |  | C_29_H_26_N_2_O_6_ | 498.1791 (-1.11) | 497, 237 |  | 4,4′-[1,3-Propanediylbis(oxy)]bis-, 1,1′-bis(4-aminobenzoate)-phenol | LJ48 |
| 4 | 2.386 | 209 | 305.0795 |  | C_15_H_10_N_6_O_2_ | 306.0865 (0.39) | 305, 237 |  | N-[2-(5-Pyrimidinyl)-2*H*-indazol-4-yl]-3-isoxazolecarboxamide | LJ48, LJ24, LJ12 |
| 5 | 2.455 | 210, 285 |  | 170.0812 | C_8_H_11_NO_3_ | 169.0739 (-0.22) |  | 134, 124, 106, 77 | 2-(4-Amino-2-hydroxyphenoxy)-ethanol | LJ24 |
| 6 | 2.455 | 210 |  | 152.0704 | C_8_H_9_NO_2_ | 151.0633 (0.87) |  | 134, 124, 106, 77 | DL-Phenylglycine | LJ24 |
| 7 | 2.468 | 210, 275 |  | 140.0678 | C_7_H_10_NO_2_ | 140.0712 (1.98) |  | 140, 112, 97, 96, 84, 56 | 2,3-Dihydro-4-methoxy-1-methyl-3-oxopyridinium | LJ48 |
| 8 | 2.468 | 207 | 179.0565 |  | C_6_H_12_O_6_ | 180.0634 (-1.83) | 71, 59 |  | L-Galactose | LJ12 |
| 9 | 2.473 | 210 |  | 203.0525 | C_8_H_10_O_6_ | 202.0477 (0.98) |  | 203, 112, 84 | 4-one, 3-hydroxy-2,5,6-tris(hydroxymethyl)-4H-Pyran | LJ12 |
| 10 | 2.473 | 210 |  | 383.1179 | C_14_H_22_O_12_ | 382.1111 (1.93) |  | 203 | (2*R*,6*R*)-5,6-dihydro-4-hydroxy-2-(hydroxymethyl)-6-methoxy-5-oxo-2*H*-pyran-3-yl-4-*O*-methyl-2-hydrate-β-D-arabino-Hexopyranosid-2-ulose | LJ12 |
| 11 | 2.555 | 210, 262 | 296.138 |  | C_12_H_19_N_5_O_4_ | 297.1437 (0.86) | 116, 89, 59 |  | L-Alanyl-L-histidyl-L-alanine | LJ12 |
| 12 | 2.555 | 206 | 179.0505 |  | C_6_H_12_O_6_ | 180.0634 (-3.69) | 71, 59 |  | L-Galactose | LJ12 |
| 13 | 2.559 | 210, 274 | 520.2192 |  | C_27_H_31_N_5_O_6_ | 521.2274 (1.83) | 288 |  | L-tryptophyl-L-prolylglycyl L-Tyrosine | LJ48 |
| 14 | 2.559 | 208, 275 | 191.0563 |  | C_7_H_12_O_6_ | 192.0634 (0.86) | 191 |  | Quinic acid | LJ48, LJ24 |
| 15 | 2.646 | 208 | 105.0197 |  | C_3_H_6_O_4_ | 106.0266 (-3.73) | 105, 75, 59 |  | 1-(hydrogen carbonate)-1,2-Ethanediol | LJ48 |
| 16 | 2.642 | 208 | 225.0619 |  | C_8_H_10_N_4_O_4_ | 226.0702 (4.30) | 71, 59 |  | N-ethyl-2-[(5-nitro-2-furanyl)methylene]-hydrazinecarboxamide | LJ12 |
| 17 | 2.906 | 260 | 151.0262 |  | C_5_H_4_N_4_O_2_ | 152.0334 (-1.24) | 108, 80 |  | Oxypurinol | LJ48 |
| 18 | 3.057 | 252 | 147.03 |  | C_5_H_8_O_5_ | 148.0372 (0.01) | 129, 103, 59 |  | 2-Hydroxyglutaric acid | LJ48, LJ24, LJ12 |
| 19 | 3.075 | 250 |  | 176.0705 | C_10_H_9_NO_2_ | 175.0633 (0.24) |  | 176, 161, 133 | 1-Acetyl-3-indolinone | LJ48 |
| 20 | 3.161 | 285 | 257.0786 |  | C_10_H_14_N_2_O_6_ | 258.0852 (-2.65) | 128 |  | 3,6-Dioxo-2,5-piperazinedipropanoic acid | LJ24, LJ12 |
| 21 | 3.49 | 220, 300, 370 |  | 168.063 | C_8_H_9_NO_3_ | 167.0582 (4.62) |  | 149, 134, 72 | D-p-Hydroxyphenylglycine | LJ24 |
| 22 | 3.508 | 255 |  | 101.0235 | C_4_H_4_O_3_ | 100.016 (-1.97) |  | 73, 55 | 4-Hydroxy-2(5*H*)-furanone | LJ48 |
| 23 | 3.508 | 255 |  | 315.0975 | C_16_H_15_N_2_O_5_ | 315.0981 (-0.05) |  | 298, 283, 271, 256 | 1-[[[2-(Acetyloxy)benzoyl]oxy]methyl]-3-(aminocarbonyl)-pyridinium | LJ48, LJ24 |
| 24 | 3.509 | 240 |  | 386.1247 | C_21_H_15_N_5_O_3_ | 385.1162 (-3.48) |  | 386, 269 | 2-Amino-N-[2,4-dihydro-3-(4-isoxazolyl)-4-oxoindeno[1,2-c]pyrazol-5-yl]-benzeneacetamide | LJ12 |
| 25 | 3.513 | 207 | 117.0197 |  | C_4_H_6_O_4_ | 118.0266 (-3.80) | 73, 55 |  | Acetoxyacetic acid | LJ48 |
| 26 | 3.513 | 212 | 214.9958 |  | C_7_H_8_N_2_O_2_S_2_ | 216.0027 (-2.77) | 117, 96, 73 |  | 2-(2-Pyridinylsulfonyl) thanethioamide | LJ48 |
| 27 | 3.599 | 210 | 194.9466 |  | C_4_H_5_ClN_2_OS_2_ | 194.9466 (-2.50) | 160, 96, 78 |  | 2-Chloro-3-thiophenesulfonimidamide | LJ48 |
| 28 | 3.599 | 210, 250 | 176.9361 |  | C_4_H_3_ClN_2_S_2_ | 177.9426 (-4.52) | 78 |  | (2-Chloro-4-thiazolyl)-methanethione | LJ48 |
| 29 | 4.011 | 204, 230, 270 | 340.1555 |  | C_20_H_23_NO_4_ | 341.1627 (0.07) | 340, 325, 310, 278, 78 |  | N-[(1,1-dimethylethoxy)carbonyl]-β-phenyl-D-phenylalanine | LJ24 |
| 30 | 4.013 | 210, 246 |  | 311.1228 | C_14_H_18_N_2_O_6_ | 310.1165 (3.38) |  | 165, 136, 120, 84 | L-Tyrosyl-L-glutamic acid | LJ48 |
| 31 | 4.033 | 210 |  | 342.1701 | C_20_H_24_NO_4_ | 342.1705 (3.15) |  | 297, 282, 254, 243, 211 | Laurifoline | LJ24, LJ12 |
| 32 | 4.021 | 210 |  | 302.1022 | C_16_H_15_NO_5_ | 301.095 (0.31) |  | 287, 259 | 2-methyl-(5,8-dihydro-7-hydroxy-6-methyl-5,8-dioxo-1-isoquinolinyl)methyl ester-2-butenoic acid | LJ48, LJ24 |
| 33 | 4.028 | 209 | 115.004 |  | C_4_H_4_O_4_ | 116.011 (-2.98) | 115, 71 |  | Maleic acid | LJ24, LJ12 |
| 34 | 4.028 | 280 |  | 267.0978 | C_12_H_14_N_2_O_5_ | 266.0903 (-0.45) |  | 120 | N-(4-Aminobenzoyl)-L-glutamic acid | LJ48 |
| 35 | 4.093 | 220 |  | 242.0812 | C_14_H_11_NO_3_ | 241.0739 (0.22) |  | 242 | [3,4-*b*]acenaphthen-10-one-a,4,5,6-Tetrahydrosuccinimido | LJ24 |
| 36 | 4.115 | 280 |  | 309.1913 | C_15_H_24_N_4_O_3_ | 308.1848 (2.54) |  | 292, 267, 250, 179, 137, 114 | L-lysyl-L-Tyrosinamide | LJ48, LJ24 |
| 37 | 4.5 | 209 | 194.9466 |  | C_4_H_5_ClN_2_OS_2_ | 194.9466 (-2.50) | 160, 96, 78 |  | 4-Chloro-2-thiophenesulfonimidamide | LJ48 |
| 38 | 4.808 | 225, 281 |  | 308.1126 | C_15_H_17_NO_6_ | 307.1056 (0.78) |  | 308, 292, 262, 248, 218, 176 | (4*R*)-5-Oxo-3-[(phenylmethoxy)carbonyl]-4-oxazolidinebutanoic acid | LJ48 |
| 39 | 4.808 | 225, 281 |  | 144.0805 | C_10_H_9_N | 143.0735 (2.52) |  | 144, 127, 117 | 2-Naphthylamine | LJ48 |
| 40 | 4.899 | 220, 280 | 216.3281 |  | C_8_H_4_Cl_2_OS | 217.936 (1.68) | 96, 78 |  | 5-ol-6,7-Dichloro-benzothiophene | LJ48 |
| 41 | 4.895 | 225 |  | 157.0831 | C_4_H_8_N_6_O | 156.076 (2.53) |  | 72, 70 | 5-(1,3,4-Oxadiazolidin-2-ylmethyl)-2*H*-tetrazole | LJ48, LJ24 |
| 42 | 4.958 | 220, 290, 380 |  | 180.1009 | C_10_H_14_NO_2_ | 180.1025 (4.21) |  | 141, 97 | 1-cyclohexyl-2,5-dioxo-3-Pyrrolidine | LJ48, LJ24 |
| 43 | 5.328 | 215 | 327.0728 |  | C_14_H_16_O_9_ | 328.0794 (-1.09) | 151, 107, 85, 97 |  | Bergenin | LJ12 |
| 44 | 5.328 | 240 |  | 135.1015 | C_6_H_14_O_3_ | 134.0943 (0.33) |  | 118, 81, 59 | Bis(2-hydroxypropyl) ether | LJ48 |
| 45 | 5.42 | 218, 280 |  | 188.0707 | C_11_H_9_NO_2_ | 187.0633 (-0.77) |  | 170, 143, 118, 91 | Indole-3-acrylic acid | LJ12 |
| 46 | 5.415 | 230, 295, 385 |  | 312.1594 | C_19_H_21_NO_3_ | 311.1511 (-0.06) |  | 255, 227, 190 | 3-(3,4-Dimethoxyphenyl)-N-(2-phenylethyl)-2-propenamide | LJ48, LJ24 |
| 47 | 5.415 | 250, 305, 375 |  | 157.0831 | C_4_H_8_N_6_O | 156.076 (-1.09) |  | 116, 96, 72, 55 | 2,4,6-Triamino-5(*H*)-pyrimidinone | LJ48 |
| 48 | 6.113 | 270 |  | 356.1493 | C_20_H_22_NO_5_ | 356.1498 (-0.18) |  | 325, 295, 285, 263, 251 | 5,6,6a,7-Tetrahydro-1,11-dihydroxy-2,10-dimethoxy-6,6-dimethyl-4-oxo-4*H*-dibenzoquinolinium | LJ24, LJ12 |
| 49 | 6.113 | 222 | 452.1106 |  | C_22_H_19_N_3_O_8_ | 453.1172 (-1.53) | 340, 96, 78 |  | N-[[2-(2,6-dioxo-3-piperidinyl)-2,3-dihydro-1,3-dioxo-1H-isoindol-4-yl]methyl]-2,3-dihydroxy-4-methoxy-benzamide | LJ48 |
| 50 | 6.196 | 215 |  | 344.1862 |  | 343.1784 (-1.51) |  | 343 | 1,2,6,7-Tetradehydro-3,11,15,16-tetramethoxy-(3β,11α)-frythrinan | LJ12 |
| 51 | 6.719 | 220 | 438.1333 |  | C_23_H_17_N_7_O_3_ | 439.1393 (-2.24) | 396, 340, 311 |  | 5-Amino-3-[2-hydroxy-5-[2-(4-nitrophenyl)diazenyl]phenyl]-1-(4-methylphenyl)-1*H*-pyrazole-4-carbonitrile | LJ48 |
| 52 | 6.82 | 220 | 259.1299 |  | C_11_H_20_N_2_O_5_ | 260.1372 (1.17) | 241, 223, 128 |  | L-Glutamyl-L-leucine | LJ48 |
| 53 | 5.761 | 230, 270 |  | 358.1643 | C_20_H_24_NO_5_ | 358.1654 (1.89) |  | 325, 295, 285, 263, 251 | 5,6,6a,7-Tetrahydro-1,4,11-trihydroxy-2,10-dimethoxy-6,6-dimethyl-4*H*-dibenzoquinolinium | LJ48, LJ24, LJ12 |
| 54 | 5.935 | 232, 290 |  | 261.1442 | C_11_H_20_N_2_O_5_ | 260.1372 (0.69) |  | 261, 244, 198, 132, 86 | γ-Glutamylleucine | LJ48 |
| 55 | 6.195 | 230, 267 |  | 282.1126 | C_17_H_15_NO_3_ | 281.1052 (-0.45) |  | 282, 176, 107 | 2-(4-Methoxyphenyl)-4-(phenylmethyl)-5(4*H*)-oxazolone | LJ48, LJ24, LJ12 |
| 56 | 6.601 | 218 |  | 307.1764 | C_14_H_26_O_7_ | 306.1679 (-4.36) |  | 177, 145, 117 | Butyl ester-β-D-galactofuranosiduronic acid | LJ24, LJ12 |
| 57 | 6.692 | 236, 290, 370 |  | 261.1442 | C_11_H_20_N_2_O_5_ | 260.1372 (3.32) |  | 261, 244, 198, 132, 86 | L-Leucyl-L-glutamic acid | LJ48 |
| 58 | 7.066 | 225 | 181.0507 |  | C_9_H_10_O_4_ | 182.0579 (0.14) | 181, 163, 135, 119, 72 |  | α,β-Dihydroxybenzenepropanoic acid | LJ48 |
| 59 | 7.066 | 298, 380 | 219.0511 |  | C_8_H_12_O_7_ | 220.0582 (0.85) | 181, 157, 111, 87 |  | 3-Deoxy-1,6-dimethyl ester-L-*threo*-2-hexulosaric acid | LJ48 |
| 60 | 7.326 | 226 | 117.0557 |  | \| C_5_H_10_O_3_ \| \| --- \| | 118.063 (2.45) | 117, 71 |  | 2-Hydroxyisovaleric acid | LJ48 |
| 61 | 7.326 | 228 | 149.0277 |  | C_5_H_10_O_3_S | 150.0351 (0.90) | 149, 101, 57 |  | 5,5-Dimethyl-2-oxide-1,3,2-dioxathiane | LJ48 |
| 62 | 7.581 | 250 |  | 314.0663 | C_18_H_19_NO_4_ | 313.1314 (-0.71) |  | 370, 270, 227, 137 | Coumarin 314 | LJ48 |
| 63 | 7.933 | 230 |  | 324.0874 | C_18_H_13_NO_5_ | 323.0974 (-4.70) |  | 324, 309, 296, 281, 253, 189 | 7-one, 6-hydroxy-5,8-dimethoxy-1-oxide7*H*-Azuleno[1,2,3-*ij*]isoquinolin | LJ12 |
| 64 | 7.755 | 240 |  | 324.0846 | C_18_H_13_NO_5_ | 323.0794 (4.54) |  | 309, 296, 281 | Oxosarcocapnidine | LJ48 |
| 65 | 8.106 | 230 |  | 326.1387 | C_19_H_19_NO_4_ | 325.1314 (3.81) |  | 328, 192 | 5,8,13,13a-tetrahydro-10-methoxy-6*H*-Benzo[*g*]-1,3-benzodioxolo[5,6-*a*]quinolizin-9-ol | LJ12 |
| 66 | 8.101 | 245 |  | 295.1289 | C_14_H_18_N_2_O_5_ | 294.1216 (-0.37) |  | 192, 166, 120, 107 | N-(N-carboxyglycyl)-N-benzyl methyl ester-alanine | LJ48, LJ24, LJ12 |
| 67 | 8.193 | 228 | 293.1144 | 295.1286 | C_14_H_18_N_2_O_5_ | 294.1216 (-1.19) | 275, 164, 128 | 192, 107 | L-Glutamyl-L-phenylalanine | LJ48, LJ24 |
| 68 | 12.001 | 270 |  | 377.1454 | C_17_H_20_N_4_O_6_ | 376.1383 (0.52) |  | 377, 243 | Riboflavin | LJ48 |
| 69 | 12.261 | 230 |  | 296.0917 | C_18_H_9_N_5_ | 295.0858 (4.44) |  | 296 | Quino[3′,2′:4,5]imidazo[1,2-*c*]quinazoline-13-carbonitrile | LJ48, LJ24, LJ12 |
| 70 | 12.526 | 260 |  | 500.1921 | C_26_H_29_NO_9_ | 499.1842 (-0.90) |  | 500, 338 | 5,7-dihydroxy-2-(4-(piperidin-1-yl)phenyl)-8-((2S,3R,4R,5S,6R)-3,4,5-trihydroxy-6-(hydroxymethyl)tetrahydro-2H-pyran-2-yl)-4H-chromen-4-one | LJ12 |
| 71 | 12.526 | 250 |  | 344.1495 | C_19_H_21_NO_5_ | 343.142 (-1.12) |  | 344, 326, 194 | α-Hydroxy-β-[[(phenylmethoxy)carbonyl]amino]-methyl ester- (αS,βS)-benzenebutanoic acid | LJ24, LJ12 |
| 72 | 13.128 | 272 | 121.0298 |  | C_7_H_6_O_2_ | 122.0368 (-3.00) | 92, 65 |  | Benzoic acid | LJ12 |
| 73 | 13.648 | 252 |  | 453.343 | C_23_H_48_O_8_ | 452.3349 (-1.27) |  | 453, 435, 417, 336, 326, 226, 210, 182, 100 | 2,2,6,6,10,10,14,14-Octamethyl-, (3R,5R,7S,9S,11R,13R)-rel-1,3,5,7,9,11,13,15-pentadecaneoctol | LJ48 |
| 74 | 13.648 | 256 |  | 475.3251 | C_25_H_46_O_8_ | 474.3193 (3.04) |  | 475, 418, 290, 151 | 2,2′-[[4,8-Bis(4-methyl-1-piperazinyl)pyrimido[5,4-d]pyrimidine-2,6-diyl]bis(methylimino)]di-ethanol | LJ48 |
| 75 | 14.086 | 244, 340 | 131.0715 |  | C_6_H_12_O_3_ | 132.0786 (0.17) | 131, 85 |  | 6-Hydroxyhexanoic acid | LJ48 |
| 76 | 14.428 | 252 |  | 398.159 | C_23_H_23_NO_6_ | 397.1525 (2.86) |  | 398, 354, 336 | 5,8,13,13a-Tetrahydro-10-methoxy-9-(2-oxiranylmethoxy)-6*H*-Benzo-1,3-benzodioxolo[5,6-*a*]quinolizine-12-carboxaldehyde | LJ48 |
| 77 | 14.606 | 244, 360 | 172.098 |  | C_8_H_15_NO_3_ | 173.1052 (-2.34) | 172, 130 |  | N-Acetyl-L-leucine | LJ48 |
| 78 | 14.953 | 250, 310, 370 |  | 386.1597 | C_21_H_23_NO_6_ | 385.1525 (-0.64) |  | 386, 325, 222 | 12-Methanol-5,8,13,13a-tetrahydro-11-hydroxy-10,14-dimethoxy-, (13aS)-6*H*-benzo[*g*]-1,3-benzodioxolo[5,6-*a*]quinolizine | LJ24, LJ12 |
| 79 | 15.213 | 242, 350 | 131.0715 |  | C_6_H_12_O_3_ | 132.0786 (0.17) | 131, 85 |  | 2-Hydroxyisocaproic acid | LJ48 |
| 80 | 20.153 | 280, 343 | 165.0555 |  | C_9_H_10_O_3_ | 166.063 (1.97) | 165, 147, 119, 103, 72 |  | β-Hydroxybenzenepropanoic acid | LJ48 |
| 81 | 21.935 | 270 |  | 392.1468 | C_23_H_22_NO_5_ | 392.1498 (4.48) |  | 392 | α-(2,6-Dimethylphenoxy)-9,10-dihydro-α-hydroperoxy-9-hydroxy-10-methyl-9-acridinemethanol | LJ48, LJ24 |
| 82 | 34.279 | 252, 345 |  | 388.1758 | C_21_H_25_NO_6_ | 387.1682 (-1.25) |  | 388, 370, 340, 224, 206, 165 | N-carboxy-N-benzyl 2-(benzyloxy)-3-hydroxypropyl ester, (R)-L-Alanine | LJ12 |
| 83 | 34.193 | 240 |  | 372.1805 | C_21_H_25_NO_5_ | 371.1733 (0.42) |  | 372, 354 | Capaurine | LJ12 |
| 84 | 55.768 | 270 |  | 346.2946 | C_19_H_39_NO_4_ | 345.2879 (1.80) |  | 346, 256 | 2-Hexyl-3-hydroxy-N-[2-hydroxy-1-(hydroxymethyl)ethyl]-decanamide | LJ48 |

LJ48, LJ24, and LJ12 mean *Lactobacillus johnsonii* cultured with YTA for 48 h, 24h, and 12h.


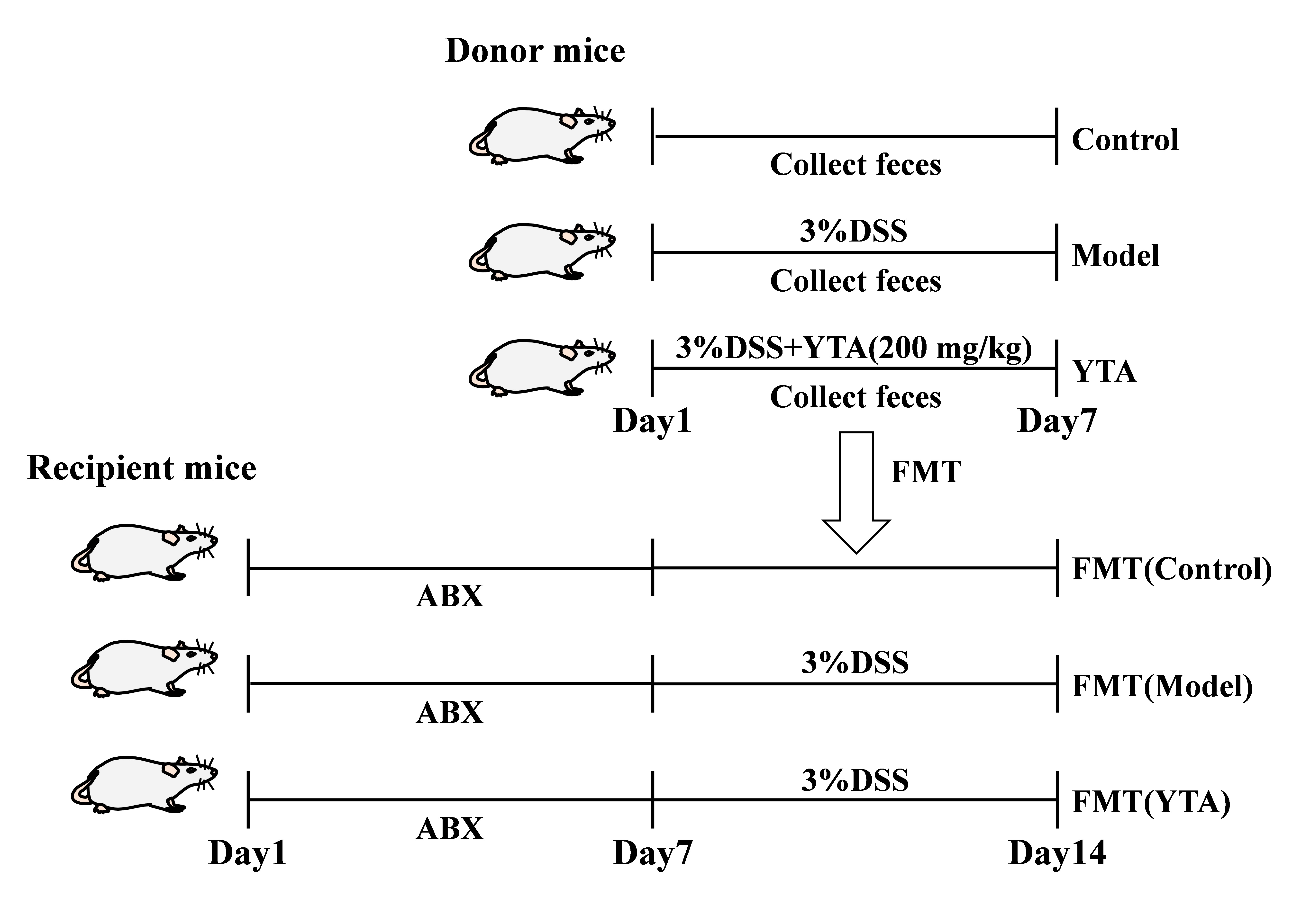
Figure S1. Schematic illustration of fecal microbiota transfer (FMT) to colitis mice.


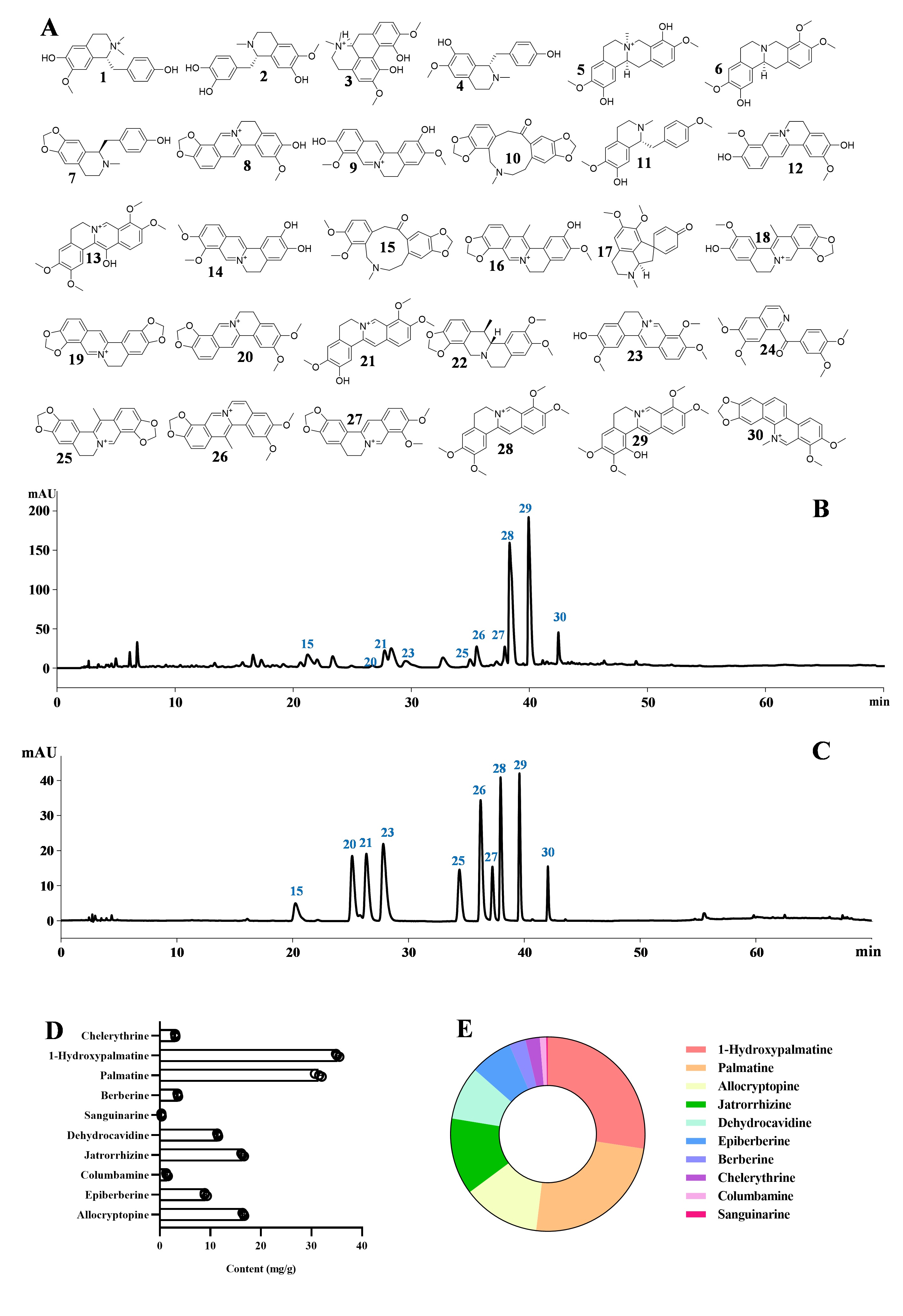
Figure S2. The analysis of chemical constitutes in YTA. (A) 30 structural formulas of compounds. (B) Liquid chromatography chromatogram of YTA at 285 nm. (C) Liquid chromatography chromatogram of mixed standard samples at 285 nm. (D) The content of main constitutes in YTA. (E) The distribution of main constitutes in YTA. (15) Allocryptopine, (20) Epiberberine, (21) Columbamine, (23) Jatrorrhizine, (25) Dehydrocavidine, (26) Sanguinarine, (27) Berberine, (28) Palmatine, (29) 1-Hydroxypalmatine, (30) Chelerythrine. (A)-(E) n = 3 per group. Mean values ± SD are presented.


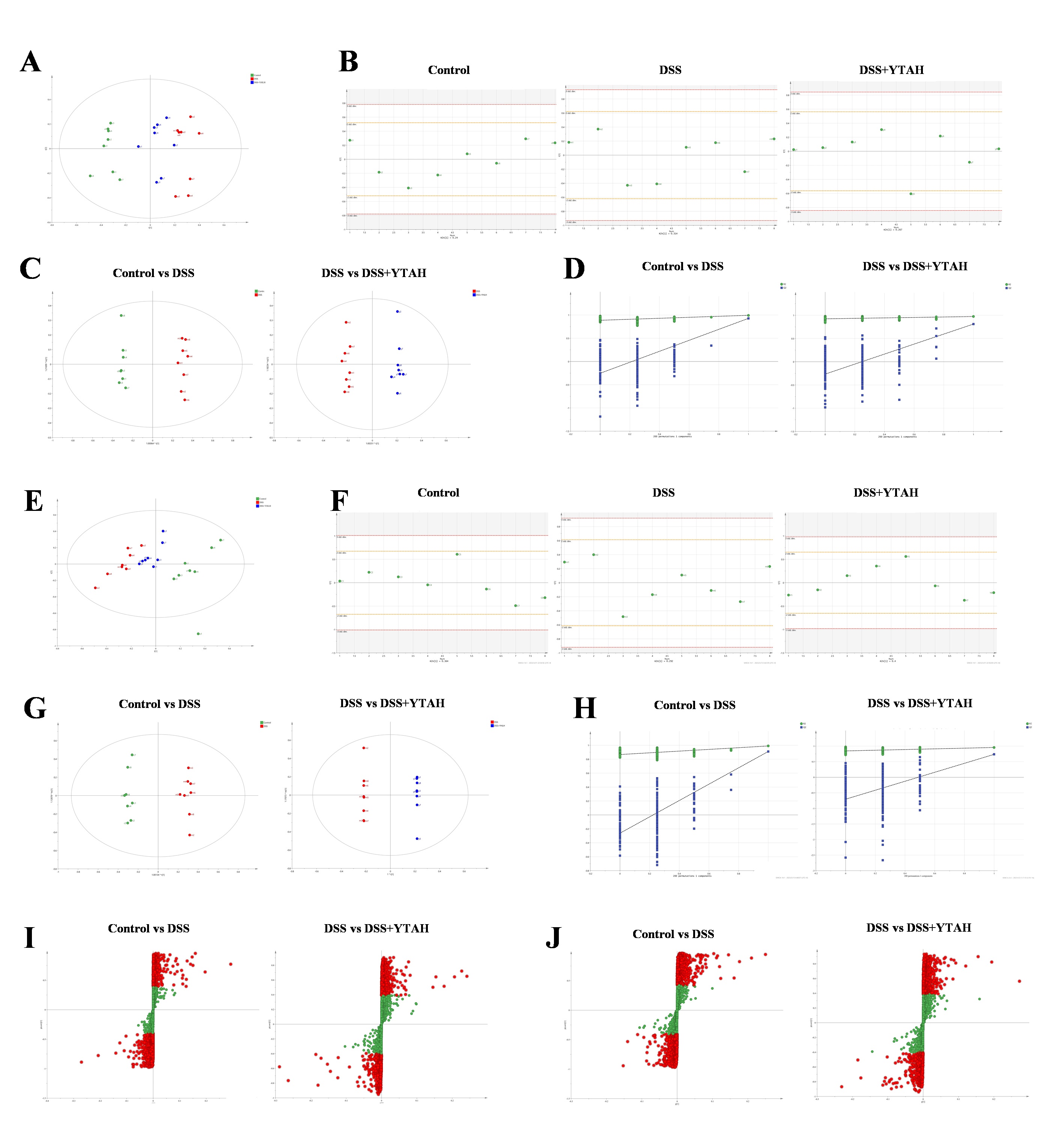
Figure S3. Colon untargeted metabolomics in DSS-induced colitis mice. (A) PCA-X analysis, (B) PCA-class, (C) OPLS-DA, (D) Permutation analysis, (I) S-plots analysis in positive mode. (E) PCA-X, (F) PCA-class, (G) OPLS-DA, (H) Permutation analysis, (J) S-plots analysis in negative mode. (A)-(J) n = 8 per group.


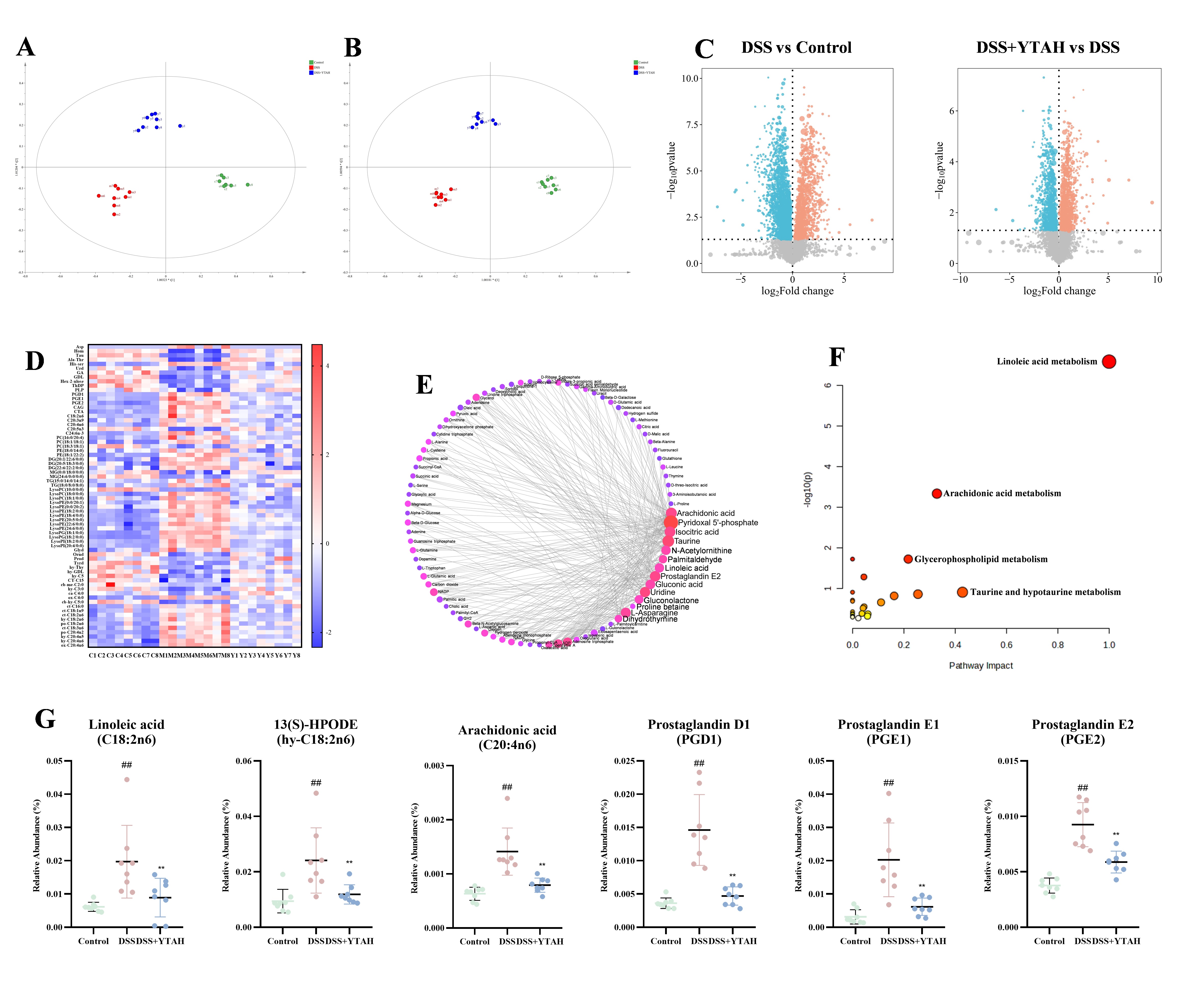
Figure S4. Serum metabolomics analysis of YTA’s therapeutic effect on DSS-induced colitis in mice. (A) OPLSDA analysis in positive mode. (B) OPLSDA analysis in negative mode. (C) Volcano plot of metabolites. (D) Heat map analysis of significant differential metabolites. (E) Network analysis of significant differential metabolites. (F) Pathway enrichment of significant differential metabolites. (G) Abundance of metabolites with a dominant position in both KEGG analysis and network analysis. (A)-(G) n = 8 per group. *P* values in (C) were calculated using t tests, *P* values in others were calculated using One-Way ANOVA, ^##^*P* < 0.01 compared with control, ^**^*P* < 0.01 and **P* < 0.05 compared with DSS.


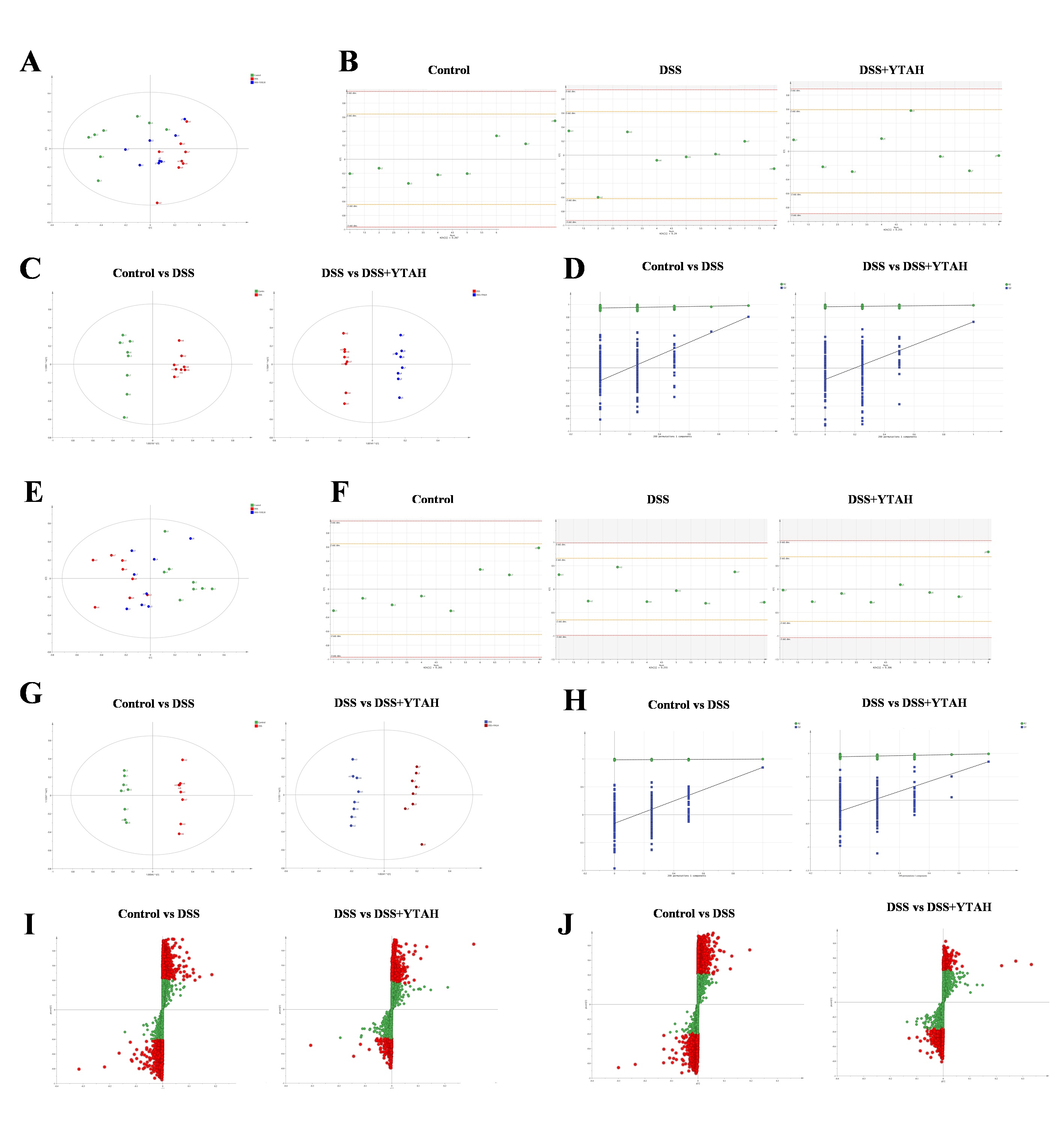
Figure S5. Serum untargeted metabolomics in DSS-induced colitis mice. (A) PCA-X, (B) PCA-class, (C) OPLS-DA, (D) Permutation analysis, (I) S-plots analysis in positive mode. (E) PCA-X, (F) PCA-class, (G) OPLS-DA, (H) Permutation analysis, (J) S-plots analysis in negative mode. (A)-(J) n = 8 per group.


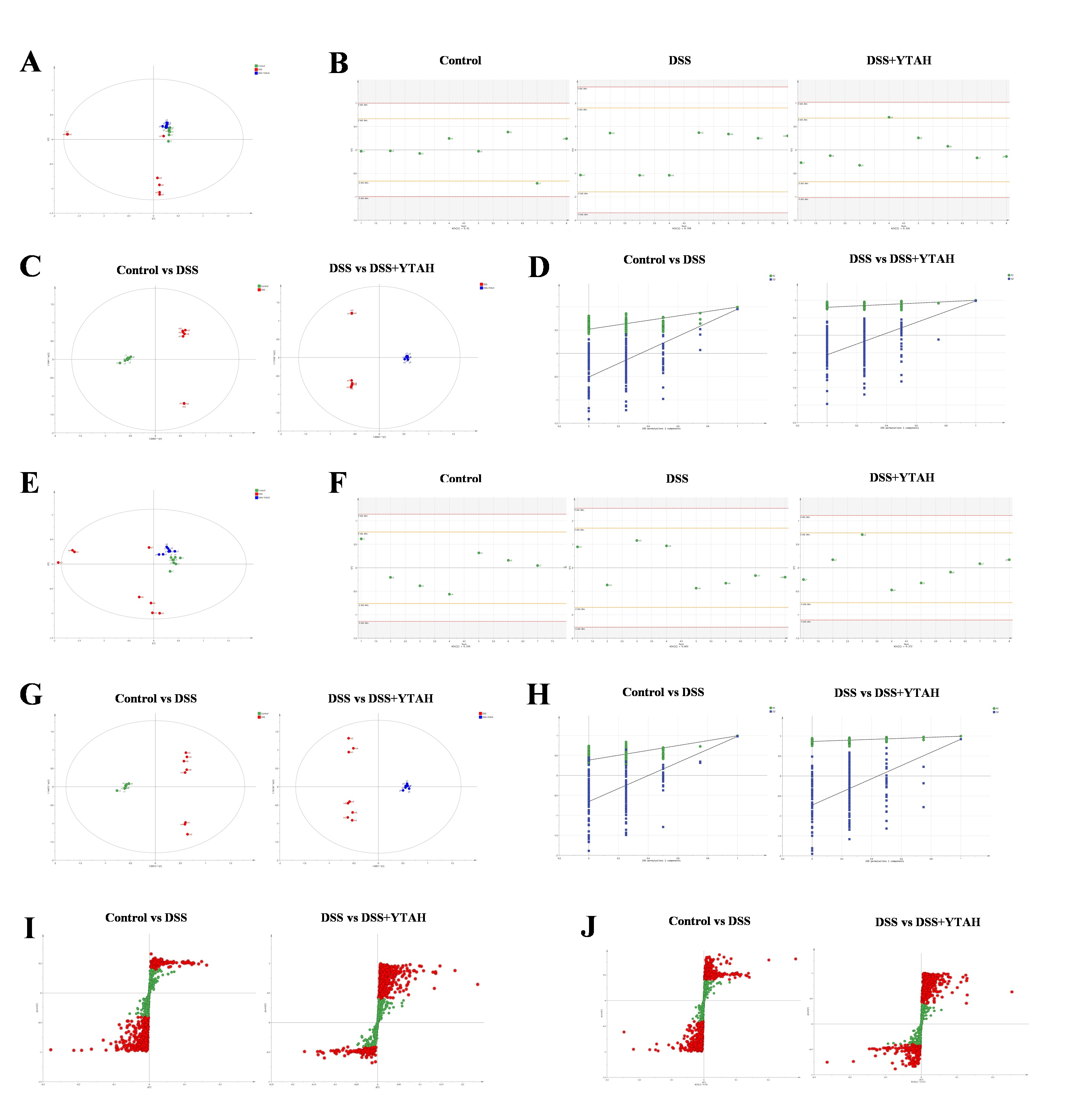
Figure S6. Fecal untargeted metabolomics in DSS-induced colitis mice. (A) PCA-X, (B) PCA-class, (C) OPLS-DA, (D) Permutation, (I) S-plots analysis in positive mode. (E) PCA-X, (F) PCA-class, (G) OPLS-DA, (H) Permutation analysis, (J) S-plots analysis in negative mode. (A)-(J) n = 8 per group.


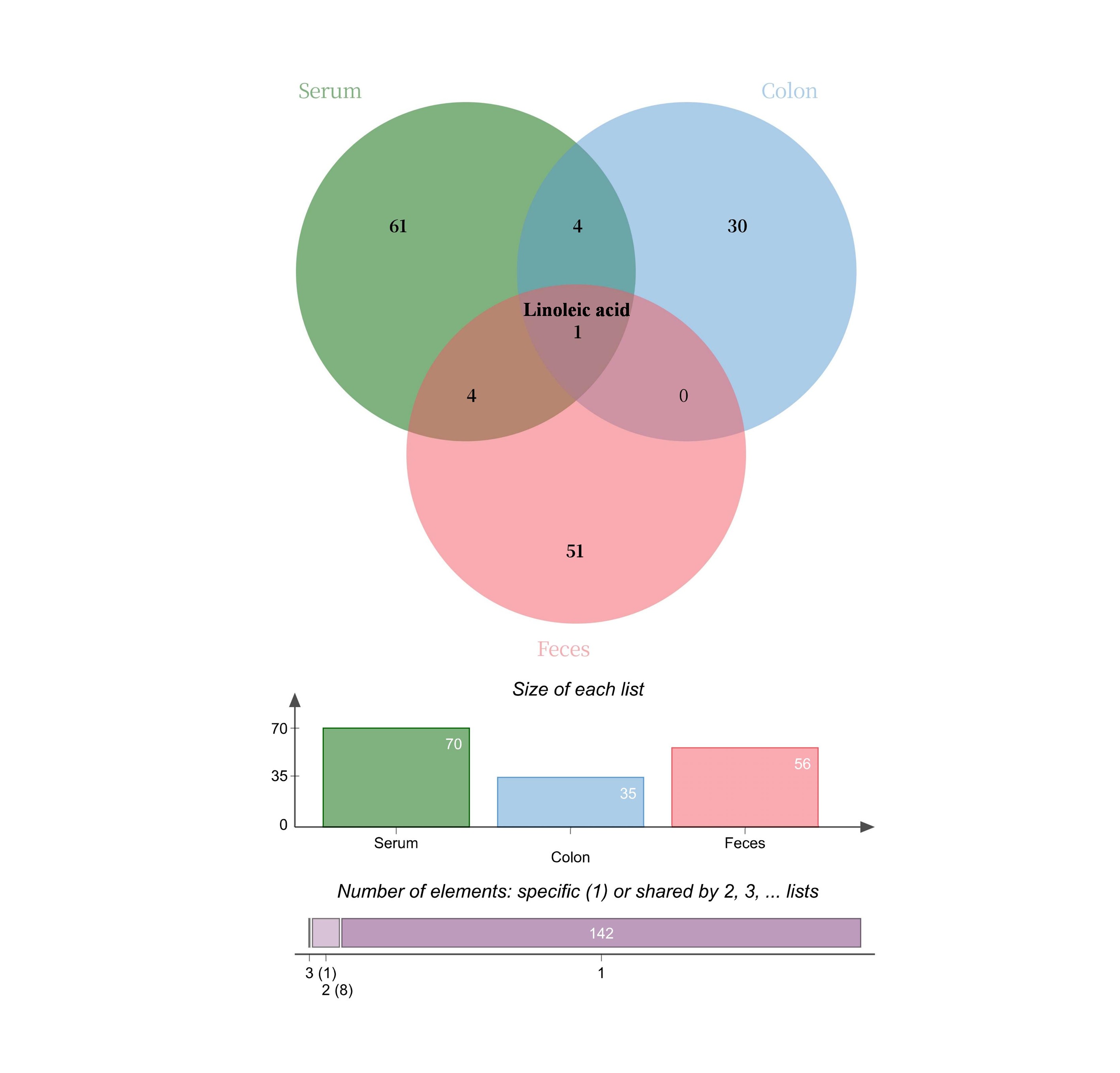
Figure S7. Venn diagram of significant differential metabolites in different metabolic profiles.


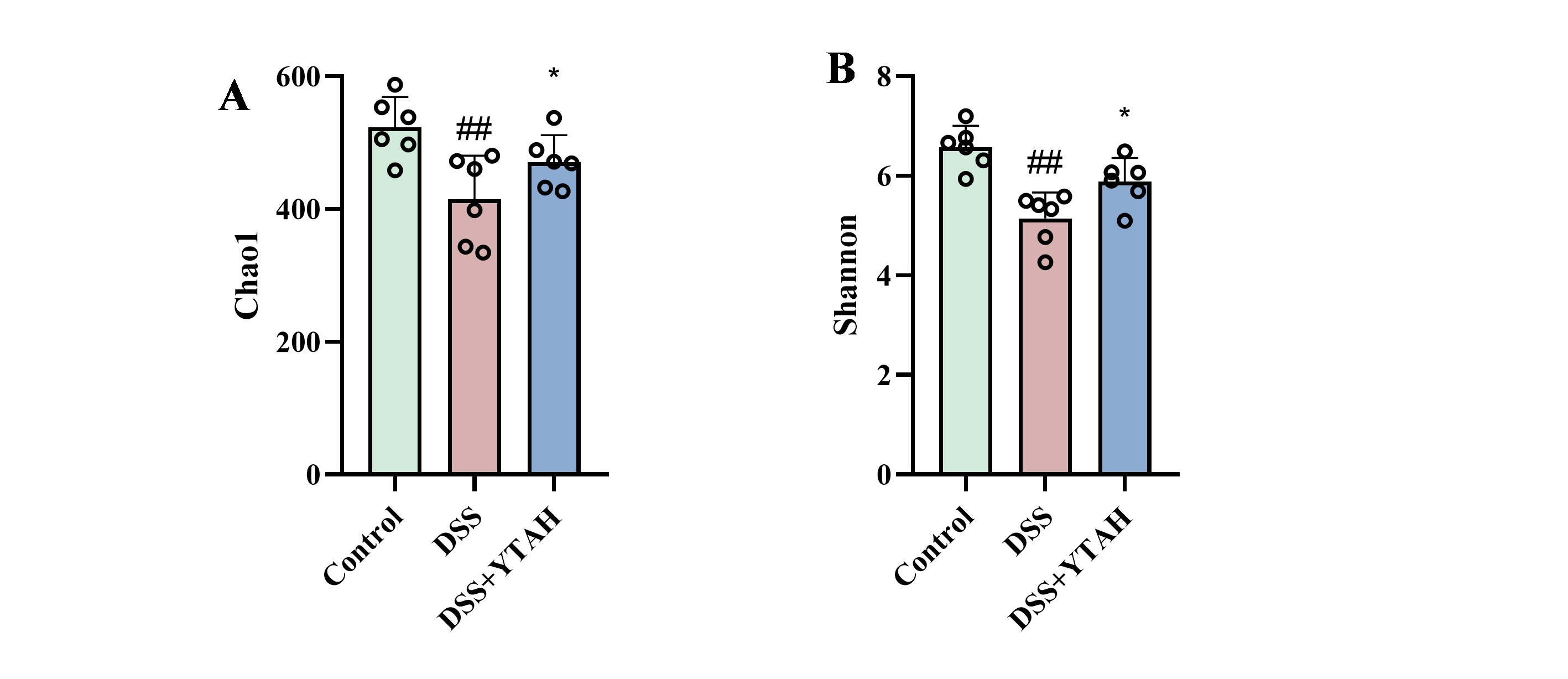
Figure S8. 16S-rRNA sequencing of feces microbiota in DSS-induced colitis mice. (A) Chao1 analysis. (B) Shannon analysis. (A) and (B) n = 6 per group. Mean values ± SD are presented. *P* values were calculated using One-Way ANOVA, ^##^*P* < 0.01 compared with control, ^**^*P* < 0.01 and **P* < 0.05 compared with DSS.


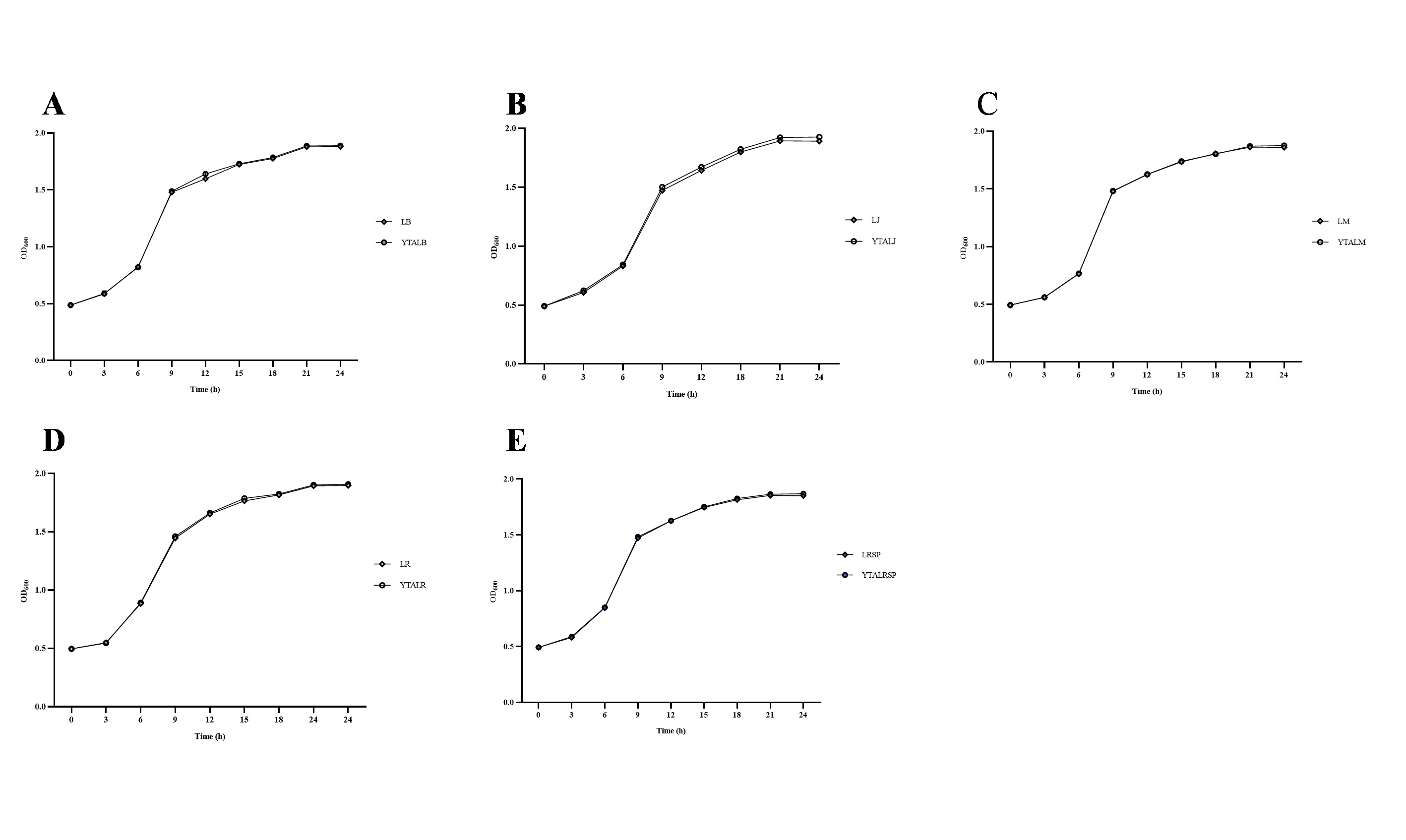
Figure S9. The growth curve of five *Lactobacillus* spp. (A) *Limosilactobacillus balticus* (LB). (B) *Lactobacillus johnsonii* (LJ). (C) *Ligilactobacillus murinus* (LM). (D) *Limosilactobacillus reuteri* (LR). (E) *Limosilactobacillus reuteri* subsp. *Reuteri* (LRSP). (A)-(E) n = 3 per group. Mean values ± SD are presented.


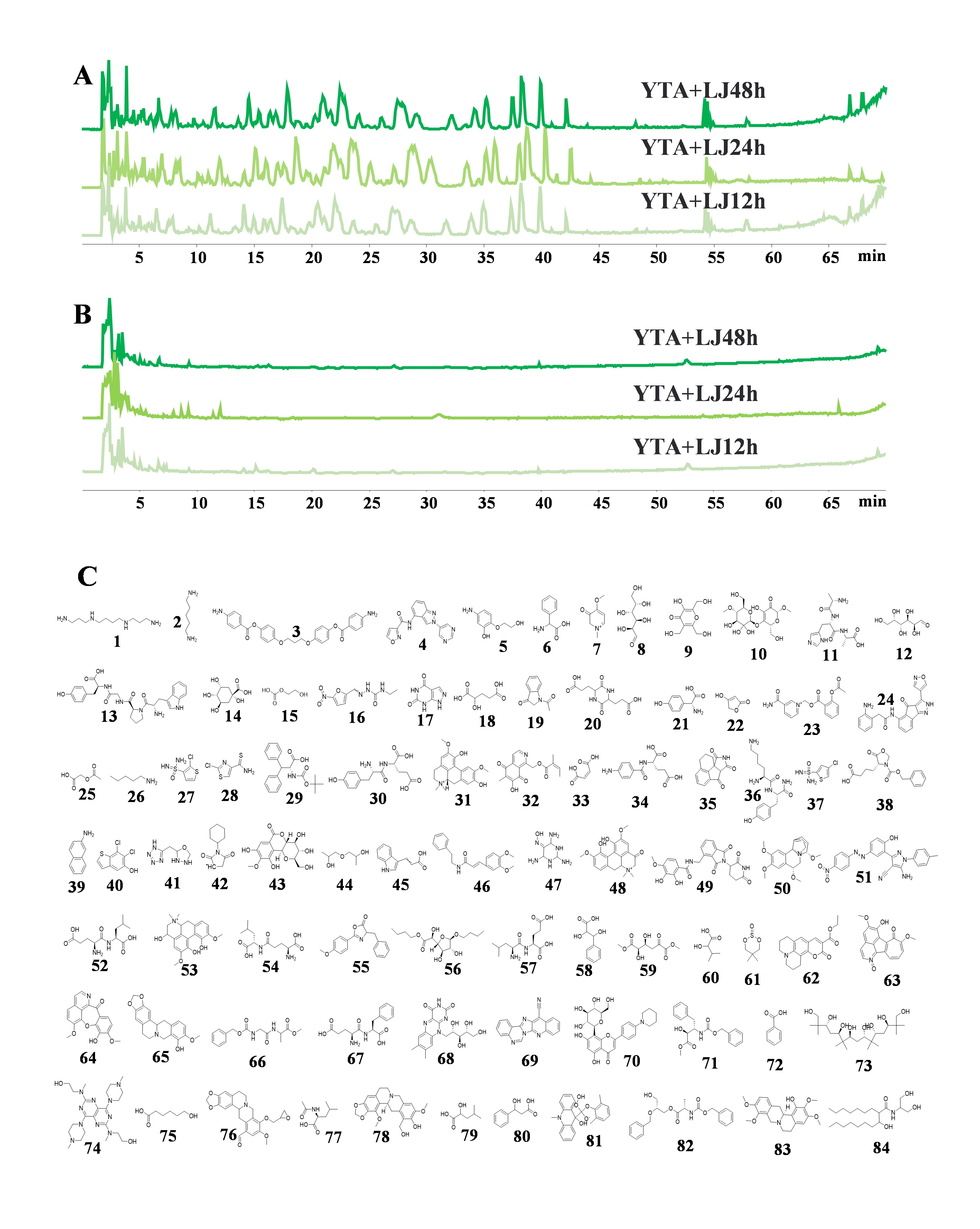
Figure S10. *L. johnsonii* cultured with YTA *in vitro*. (A) Total ion chromatogram in positive modes. (B) Total ion chromatogram in negative modes. (C) 84 structural formulas of compounds.


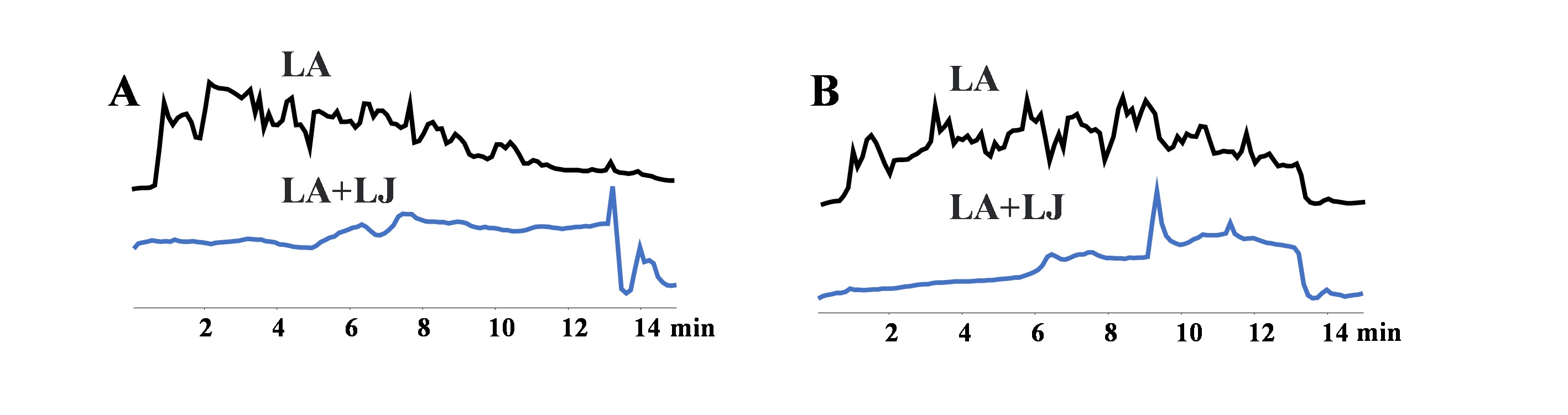
Figure S11. *L. johnsonii* decomposed LA *in vitro*. (A) Total ion chromatograms in positive modes. (B) Total ion chromatograms in negative modes.
